# Supplementary material for: The MALDI-TOF E2/E3 Ligase Assay as Universal Tool for Drug Discovery in the Ubiquitin Pathway
Source: Cell Chem Biol. 2018 Sep 20;25(9):1117–1127.e4. doi: 10.1016/j.chembiol.2018.06.004 (PMC6162346; doi:10.1016/j.chembiol.2018.06.004)
Supplement: Document S1. Figures S1–S6 and Tables S1–S5 [file mmc1.pdf]

Cell Chemical Biology, Volume *25*

Supplemental Information

The MALDI-TOF E2/E3 Ligase Assay as Universal Tool for Drug Discovery in the Ubiquitin Pathway

Virginia De Cesare, Clare Johnson, Victoria Barlow, James Hastie, Axel Knebel, and Matthias Trost

Supplementary Data

Table S1: Initial reaction/Instantaneous rate at 5 min of MDM2, ITCH and HOIP (*related to Figure 1*)

Instantaneous Rate at *t* = 5 min

**µM Ub**	**12.5**	**6.25**	**3.125**	
MDM2	0.61	0.24	0.30	µmol/min
ITCH	0.41	0.34	0.30	µmol/min
HOIP	1.85	1.28	0.94	µmol/min

Table S2: List of E1 Activating enzymes, E2 conjugating enzymes and E3 ligases used in this study (*related to Figure 2 and STAR methods*)

		Name	Uniprot Accession Number	Alternative Name	Tag	Domain	Host	E2/E3 active pair	Activity
E1	1	UBE1	P22314	-	His	full length	*S. frugiperda*	-	-
	2	UBA6	A0AVT1	-	His	full length	*S. frugiperda*	-	-
E2 Conjugating Enzyme	3	UBE2A	P49459	-	His	2-152	*E. coli*	-	-
	4	UBE2B	P63146	-	His	full length	*E. coli*	-	-
	5	UBE2C	O00762	UbcH10	-	full length	*E. coli*	ITCH	Low
	6	UBE2D1	P51668	UbcH5a	-	full length	*E. coli*	MDM2-ITCH-HOIP	High
	7	UBE2D2	P62837	UbcH5b	His	2-147	*E. coli*	MDM2-ITCH-HOIP	High
	8	UBE2D3	P61077	UbcH5c	His	2-147	*E. coli*	MDM2-ITCH-HOIP	Moderate-High
	9	UBE2D4	Q9Y2X8	UbcH5D	-	full length	*E. coli*	MDM2-ITCH-HOIP	Moderate-High
	10	UBE2E1	P51965	UbcH6	His	full length	*E. coli*	MDM2-ITCH-HOIP	Low-Moderate
	11	UBE2E2	Q96LR5	UbcH8	His	full length	*E. coli*	MDM2-HOIP	Moderate
	12	UBE2E3	Q969T4	UbcH9	His	full length	*E. coli*	MDM2-HOIP	Moderate
	13	UBE2G1	P62253	Ubc7	His	full length	*E. coli*	-	-
	14	UBE2G2	P60604	-	His	full length	*E. coli*	-	-
	15	UBE2H	P62256	Ubc8	His	full length	*E. coli*	-	-
	16	UBE2J1	Q9Y385	Ubc6e	His	full length	*E. coli*	-	-
	17	UBE2J2	Q8N2K1	-	-	full length	*E. coli*	-	-
	18	UBE2K	P61086	Ubc1	His	full length	*E. coli*	MDM2-ITCH-HOIP	Low
	19	UBE2L3	P68036	UbcH7	-	full length	*E. coli*	ITCH-HOIP	Moderate-High
	20	UBE2N	P61088	Ubc13	His	full length	*E. coli*	-	-
	21	UBE2O	Q9C0C9	E2-230K	His	552-1292	*E. coli*	-	Basal Activity
	22	UBE2Q1	Q7Z7E8	UBE2Q	His	full length	*E. coli*	-	Basal Activity
	23	UBE2Q2	Q8WVN8	-	His	full length	*E. coli*	-	Basal Activity
	24	UBE2R1	P49427	-	His	2 – 236	*E. coli*	-	-
	25	UBE2R2	Q712K3	Ubc3B	-	full length	*E. coli*	-	-
	26	UBE2S	Q16763	E2-EPF	His	full length	*E. coli*	-	-
	27	UBE2T	Q9NPD8	HSPC150	His	full length	*E. coli*	-	-
	28	UBE2V1	Q13404	Uev1A	His	full length	*E. coli*	-	-
	29	UBE2W	Q96B02	-	His	full length	*E. coli*	-	-
	30	UBE2Z	Q9H832	USE1	His	full length	*E. coli*	-	-
E3 Ligase	31	MDM2	Q00987	-	GST	7 - 497	*E. coli*	-	-
	32	ITCH	Q96J02	-	GST	full length	*E. coli*	-	-
	33	HOIP	Q96EP0	-	-	697-1072	*E. coli*	-	-

E. coli, *Escherichia coli*; *S. frugiperda*, *Spodoptera frugiperda*; *Low (≤50% Activity after 2h incubation time); Moderate (≥50% and ≤80% Activity after 2h incubation time); High 100% Activity after 2h Incubation time

Table S3| IC50 calculation of six E1, E2 or E3 inhibitors (*related to Figure 4*)

IC50 (µM)

	PR619	Nutlin3A	Gliotoxin	BAY117082	PYR41	Compound1
MDM2	0.6	Ambiguous	0.5	2.4	3.1	1.2
ITCH	0.4	Ambiguous	30.6	25.9	11.3	2.2
HOIP	0.2	Ambiguous	2.8	2.9	5.7	2.3

Table S4| Hill Slopes values for calculated IC50s (*related to Figure 4*)

Hill Slope Values

	PR619	Nutlin3A	Gliotoxin	BAY117082	PYR41	Compound1
MDM2	1.89	-	2.18	3.5	2.23	0.67
ITCH	3.14	-	5.12	1.23	1.22	0.78
HOIP	7.8	-	2.01	2.48	3.73	1.14

Table S5| Z-Prime scores (*related to Figure 5*)

	Plate				
	A	B	C	D	E
MDM2	0.64	0.57	0.63	0.62	0.55
ITCH	0.67	0.64	0.73	0.83	0.79
HOIP	0.60	0.56	0.59	0.67	0.62

Figure S1

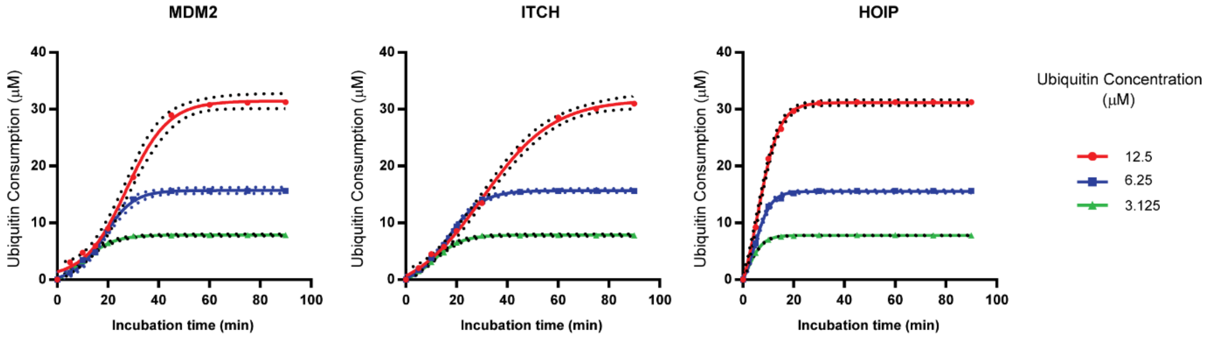

Figure S1: Substrate consumption over time of MDM2, ITCH and HOIP. *(*related to Figure 1*)*
Rate of disappearance of ubiquitin at different starting concentrations were measured for MDM2, ITCH and HOIP. Ubiquitin consumption (Ubiquitin µM t2 - Ubiquitin µM t1)) was plotted over time. Data are represented as mean +/- standard deviation.

Figure S2

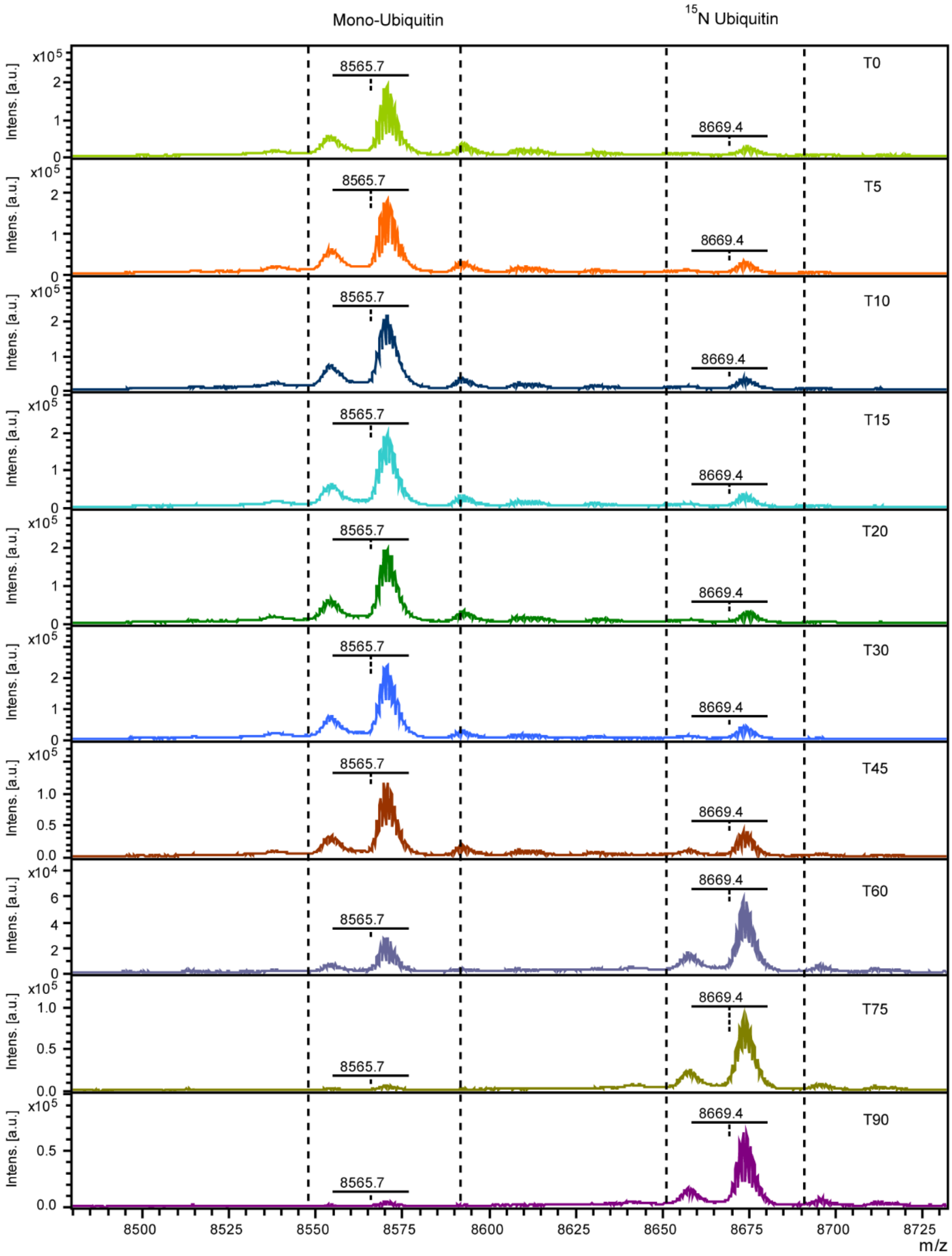

Figure S2: E2-E3 MALDI-TOF assay representative spectra (*related to Figure 1*). MALDI-TOF spectra of MDM2 activity at different time points (indicated as T0, T5 etc.) are reported. MDM2 was incubated with 50 nM E1, 250 nM E2D1, 1 mM ATP, 12.5 µM ubiquitin at 37°C and terminated at the indicated time points by addition of 2.5 µl of 10% (v/v) trifluoroacetic acid (TFA). The samples were spotted and analysed by high mass accuracy MALDI TOF MS as indicated in Material and Methods.

Figure S3

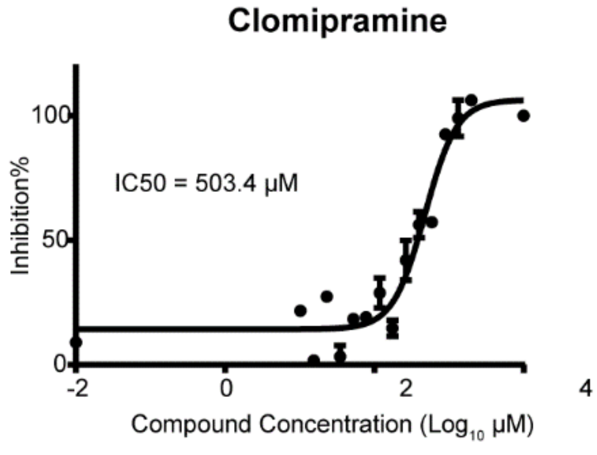

Figure S3: Clomipramine inhibits ITCH at high concentration *(related to Figure 4).* Clomipramine was tested against ITCH in a dose-response curve with final concentration values between 2mM and 7 µM. The results indicated an IC50 of 503.4 µM. Data are represented as mean +/- standard deviation.

Figure S4

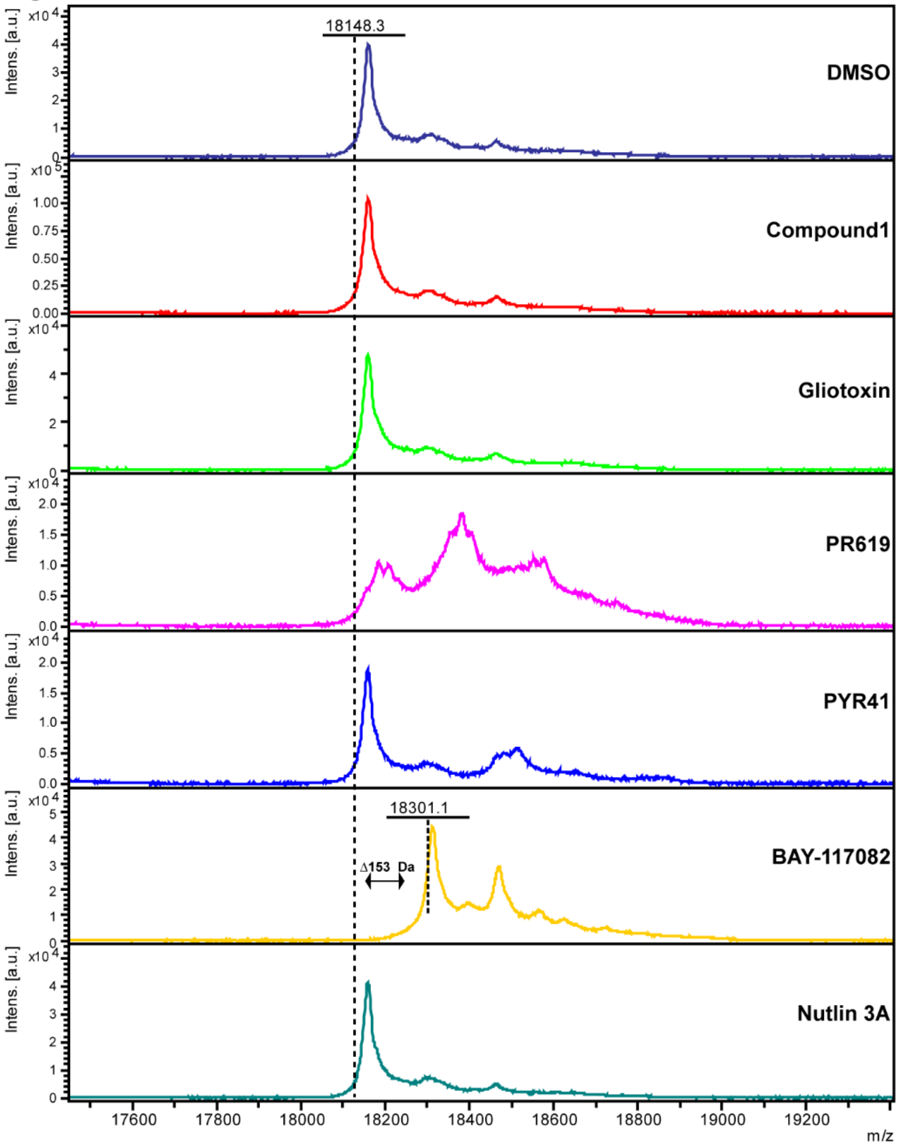

Figure S4:Covalent modifications of E2 enzymes by inhibitors *(related to Figure 4)*.
UBE2L3 (3 µM) was incubated for 30 min at room temperature with or without the indicated inhibitors (100 µM) and subjected to MALDI–TOF-MS. Incubation with BAY 11-7082 increased the molecular mass of UBE2L3 by 153 Da. PR619 extensively modified UBE2L3 indicated several PR619 adducts.

Figure S5

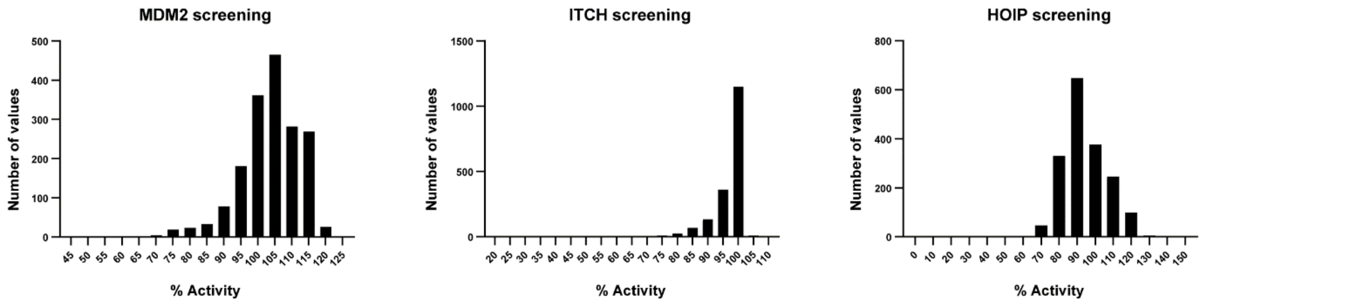

Figure S5: Frequency distribution of HTS data of MDM2, ITCH and HOIP *(related to Figure 5)*. Bars report number of values falling within the reported % Activity.

Figure S6

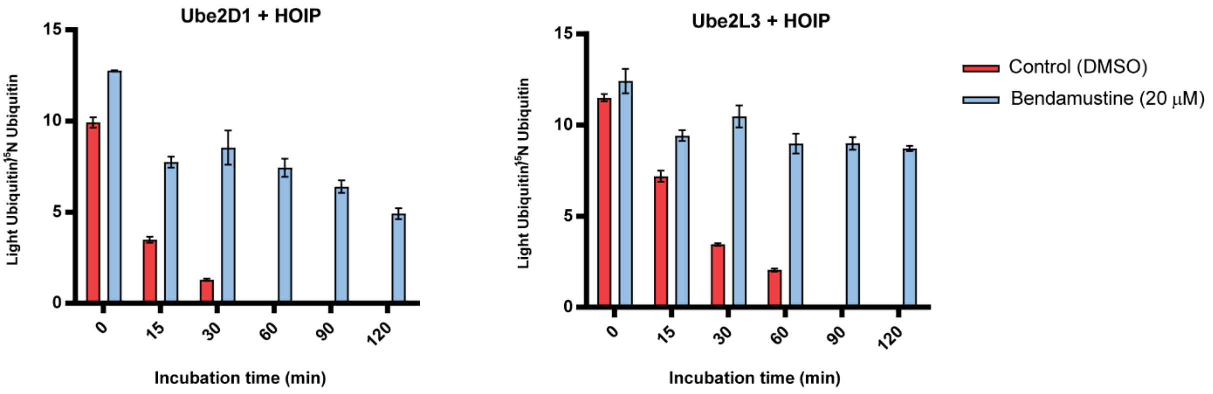

Figure S6: Bendamustine inhibits HOIP independently from the E2 conjugating enzyme *(related to Figure 6).*
Bendamustine (20 µM) was tested against HOIP coupled with Ube2D1 and Ube2L3 over time. Bendamustine inhibits HOIP independently from the E2 in use. Data are represented as mean +/- standard deviation.
