# Supplementary material for: The MALDI-TOF E2/E3 Ligase Assay as Universal Tool for Drug Discovery in the Ubiquitin Pathway
Source: Cell Chem Biol. 2018 Sep 20;25(9):1117–1127.e4. doi: 10.1016/j.chembiol.2018.06.004 (PMC6162346; doi:10.1016/j.chembiol.2018.06.004)
Supplement: Document S2. Article plus Supplemental Information [file mmc2.pdf]

Article

Cell Chemical Biology

The MALDI-TOF E2/E3 Ligase Assay as Universal Tool for Drug Discovery in the Ubiquitin Pathway

Graphical Abstract

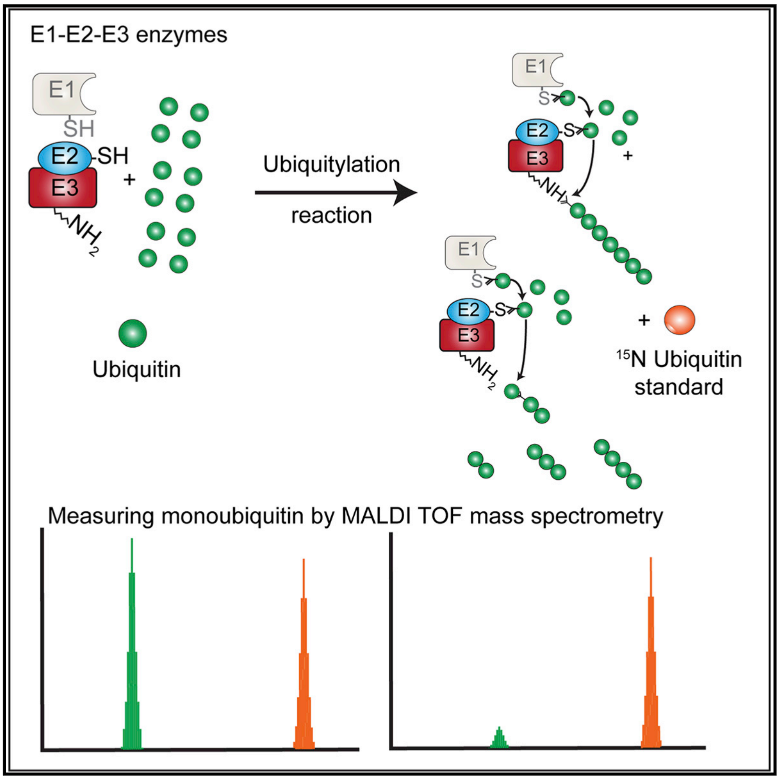

Authors

Virginia De Cesare, Clare Johnson, Victoria Barlow, James Hastie, Axel Knebel, Matthias Trost

Correspondence

v.decesare@dundee.ac.uk (V.D.C.),
matthias.trost@ncl.ac.uk (M.T.)

In Brief

Due to their role in many diseases, there is a need to identify drug candidates for enzymes of the ubiquitin system in robust and high-throughput assays. Here we report as label-free high-throughput screening assay for ubiquitin E2 conjugating enzymes and E3 ligases based on MALDI-TOF mass spectrometry.

Highlights

- We have developed a high-throughput MALDI-TOF assay for E2/E3 enzymes
- It allows screening compound libraries without chemical or fluorescent probes
- We tested the screen on three disease-relevant E3 ligases: MDM2, ITCH, and HOIP
- We performed a proof-of-concept high-throughput screen against 1,430 compounds

De Cesare et al., 2018, Cell Chemical Biology *25*, 1117–1127
September 20, 2018 © 2018 The Authors. Published by Elsevier Ltd.
https://doi.org/10.1016/j.chembiol.2018.06.004

Check for updates

CellPress

Cell Chemical Biology
Article

The MALDI-TOF E2/E3 Ligase Assay as Universal Tool for Drug Discovery in the Ubiquitin Pathway

Virginia De Cesare,[1,*] Clare Johnson,[2] Victoria Barlow,[2] James Hastie,[2] Axel Knebel,[1] and Matthias Trost[1,3,4,*]
[1]MRC Protein Phosphorylation and Ubiquitylation Unit, University of Dundee, Dow St, Dundee DD1 5EH, Scotland, UK
[2]MRC Protein Phosphorylation and Ubiquitylation Unit Reagents and Services, University of Dundee, Dow St, Dundee DD1 5EH, Scotland, UK
[3]Institute for Cell and Molecular Biosciences, Newcastle University, Framlington Place, Newcastle-upon-Tyne NE2 1HH, UK
[4]Lead Contact
*Correspondence: v.decesare@dundee.ac.uk (V.D.C.), matthias.trost@ncl.ac.uk (M.T.)
https://doi.org/10.1016/j.chembiol.2018.06.004

SUMMARY

Due to their role in many diseases, enzymes of the ubiquitin system have recently become interesting drug targets. Despite efforts, primary screenings of compound libraries targeting E2 enzymes and E3 ligases have been strongly limited by the lack of robust and fast high-throughput assays. Here we report a label-free high-throughput screening assay for ubiquitin E2 conjugating enzymes and E3 ligases based on MALDI-TOF mass spectrometry. The MALDI-TOF E2/E3 assay allows testing E2 enzymes and E3 ligases for their ubiquitin transfer activity, identifying E2/E3 active pairs, inhibitor potency and specificity and screening compound libraries *in vitro* without chemical or fluorescent probes. We demonstrate that the MALDI-TOF E2/E3 assay is a universal tool for drug discovery screening in the ubiquitin pathway as it is suitable for working with all E3 ligase families and requires a reduced amount of reagents, compared with standard biochemical assays.

INTRODUCTION

Ubiquitylation is a post-translational modification which impacts almost every biological process in the cell. Dysregulation of the ubiquitylation pathway is associated with several diseases, including cancer, neurodegenerative disorders, and immunological dysfunctions. Single ubiquitin moieties or polyubiquitin chains are added to the substrate by the combined action of three different classes of enzymes: the E1 activating enzymes, the E2s conjugating enzymes, and the E3 ligase enzymes (Pickart, 2001). In the first step, a single ubiquitin molecule is coupled to the active site of an E1 ubiquitin-activating enzyme in an ATP-dependent reaction. In the second step, the ubiquitin molecule is transferred from E1 to an E2 ubiquitin conjugating enzyme. In the final step, ubiquitin is transferred to the protein substrate in a process mediated by an E3 ubiquitin ligase, which provides a binding platform for ubiquitin-charged E2 and the substrate. Ubiquitin chain formation is highly specific and regulated by a plethora of different E2 conjugating enzymes and E3 ligases. The human genome encodes two ubiquitin-activating E1, >30 ubiquitin-specific E2, and 600–700 of E3 ligases (Kim et al., 2011). Thus, including about 100 deubiquitylating enzymes, approximately 800 ubiquitin enzymes regulate the dynamic ubiquitylation of a wide range of protein substrates (Kim et al., 2011). Within this complexity, E3 ligases are the most diverse class of enzymes in the ubiquitylation pathway as they play a central role in determining the selectivity of ubiquitin-mediated protein degradation and signaling.

E3 ligases have been associated with a number of pathogenic mechanisms. Mutations in the E3 ligases MDM2, BRCA1, TRIMs, and Parkin have been linked to multiple cancers and neurodegenerative diseases (Fakharzadeh et al., 1991; Hatakeyama, 2011; Welcsh and King, 2001), and MDM2-p53 interaction inhibitors have already been developed as a potential anticancer treatment (Shangary and Wang, 2009). This highlights the potential of E2 enzymes and E3 ligases as drug targets. Although all E3 ligases are involved in the final step of covalent ubiquitylation of target proteins, they differ in both structure and mechanism and can be classified in three main families depending on the type of E3 ligases promoting ubiquitin-protein ligation and on the presence of characteristic domains. The RING ligases bring the ubiquitin-E2 complex into the molecular vicinity of the substrate and facilitate ubiquitin transfer directly from the E2 enzyme to the substrate protein. In contrast, homologous to the E6-AP C terminus family (HECTs) covalently bind the ubiquitin via a cysteine residue in their catalytic HECT domain before shuttling it onto the target molecule. RING between RINGs (RBRs) E3 ligases were shown to use both RING- and HECT-like mechanisms where ubiquitin is initially recruited on a RING domain (RING1) then transferred to the substrate through a conserved cysteine residue in a second RING domain. The vast majority of human E3 enzymes belong to the RING family, while only 28 belong to the HECT and 14 to the RBR family of E3 ligases (Chaugule and Walden, 2016).

Due to the high attractiveness of E2 and E3 ligases as drug targets, a number of drug discovery assays have been published, based on detection by fluorescence (Dudgeon et al., 2010; Krist et al., 2016; Zhang et al., 2004), antibodies (Davydov et al., 2004; Huang et al., 2005; Kenten et al., 2005; Marblestone et al., 2010), tandem ubiquitin-binding entities (Heap et al.,

This is an open access article under the CC BY license (http://creativecommons.org/licenses/by/4.0/).

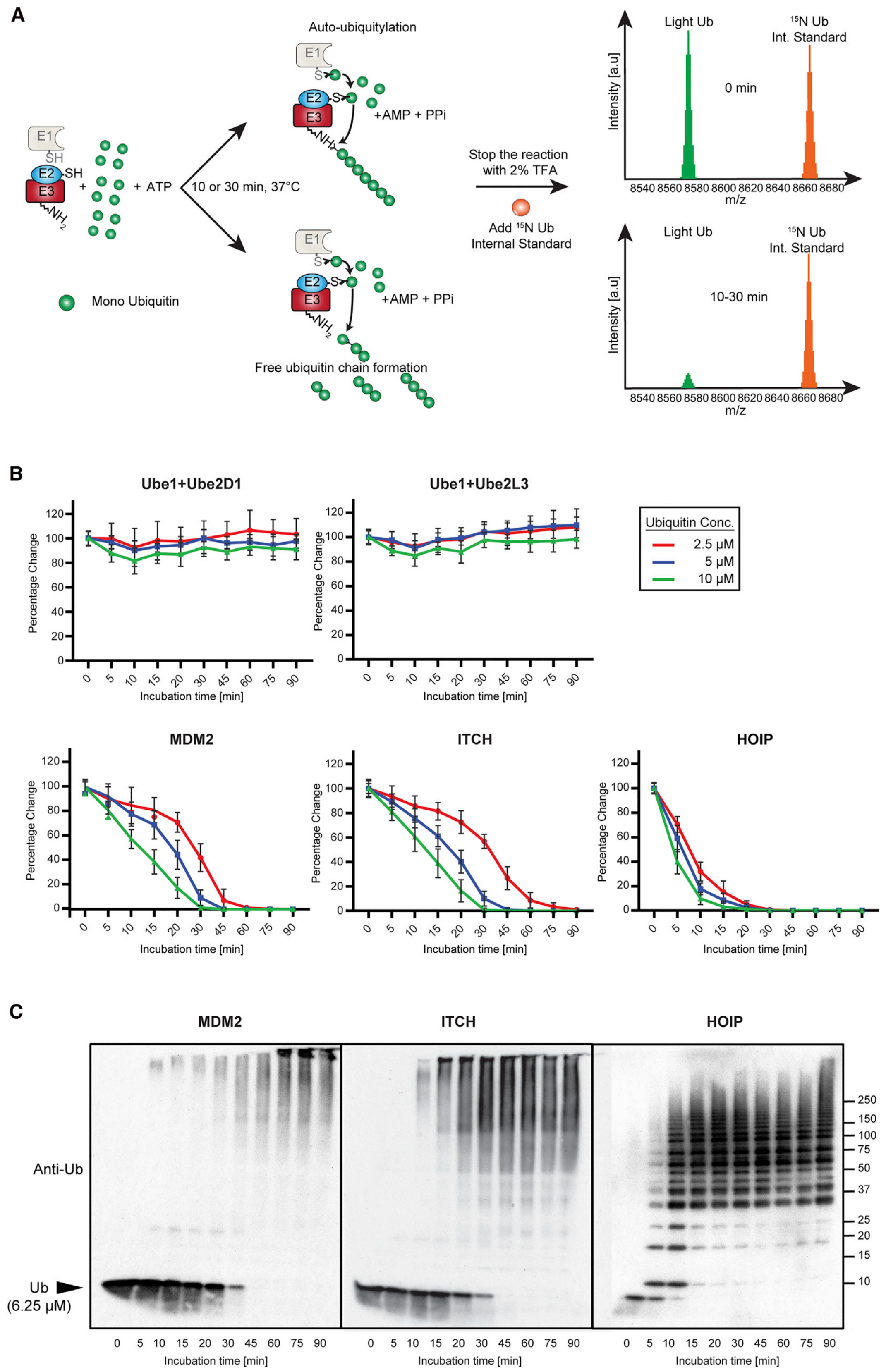

(legend on next page)

2017a; Marblestone et al., 2012), surface plasmon resonance (Regnstrom et al., 2013), or cellular and bacterial two-hybrid (Levin-Kravets et al., 2016; Maculins et al., 2016). However, many of these tools are either too expensive for very high-throughput drug discovery or potentially result in false-positive and false-negative hits due to the use of non-physiological E2/E3 ligase substrates. We have addressed this gap by developing the first *in vitro* label-free MALDI-TOF mass spectrometry-based approach to screen the activity of E2 and E3 ligases that uses unmodified mono-ubiquitin as substrate. As a proof-of-concept, we screened a collection of 1,430 US Food and Drug Administration (FDA)-approved drugs for inhibitors of a subset of three E3 ligases that are clinically relevant and belong to three different E3 ligase families. The screen shows high reproducibility and robustness, and we were able to identify a subset of 15 molecules active against the E3 ligases tested. We validated the most powerful positive hits by determining the half maximal inhibitory concentration (IC_{50}) values against their targets, confirming that bendamustine and candesartan cilexitel inhibit HOIP and MDM2, respectively, in *in vitro* conditions.

RESULTS

MALDI-TOF E2-E3 Assay Rational and Development

E2 and E3 ligase activity results in formation of free or attached polyubiquitin chains, mono-ubiquitylation, and/or multiple mono-ubiquitylation of a specific substrate. However, in absence of a specific substrate, most E3 ligases will either produce free polyubiquitin chains or undergo auto-ubiquitylation which is a mechanism thought to be responsible for the regulation of the E3 enzyme itself (de Bie and Ciechanover, 2011). Furthermore, there is some evidence that auto-ubiquitylation of E3 ligases is facilitating the recruitment of the E2 ubiquitin conjugating enzyme (Ranaweera and Yang, 2013). Auto-ubiquitylation assays or free polyubiquitin chain production have been widely used to assess the E3 ligase potential of a protein (de Bie and Ciechanover, 2011; Lorick et al., 1999). We used this property of E2 and E3 ligases to design a MALDI-TOF mass spectrometry-based high-throughput screening (HTS) method that allowed the reliable determination of activities of E2 and E3 ligase pairs by measuring the depleting intensity of mono-ubiquitin in the assay as a readout.

As proof-of-concept we used three E3 ligases belonging to different E3 families and representative of all the currently known ubiquitylation mechanisms. MDM2 is an RING-type E3 ligase which controls the stability of the transcription factor p53, a key tumor suppressor that is often found mutated in human cancers (Rivlin et al., 2011; Vogelstein et al., 2000). ITCH belongs to the HECT domain-containing E3 ligase family involved in the regulation of immunological response and cancer development (Hansen et al., 2007; Rivetti di Val Cervo et al., 2009; Rossi et al., 2009). Finally, HOIP, an RBR E3 ubiquitin ligase and member of the LUBAC (linear ubiquitin chain assembly complex). As part of the LUBAC complex, HOIP is involved in the regulation of important cellular signaling pathways that control innate immunity and inflammation through nuclear factor nuclear factor κB (NF-κB) activation and protection against tumor necrosis factor α-induced apoptosis (Kirisako et al., 2006; Tokunaga et al., 2009). HOIP is the only known E3 ligase generating linear ubiquitin chains (Ikeda et al., 2011). Because of that, fluorescent assays using C- or N-terminally labeled ubiquitin species cannot be used to form linear chains.

To determine MDM2, ITCH, and HOIP auto-ubiquitylation reaction rate and the linearity range we followed the consumption of mono-ubiquitin over time with increasing starting amount of mono-ubiquitin. We matched MDM2, ITCH, and HOIP with E2 conjugating enzymes as reported in the literature: MDM2 and ITCH were incubated with E2D1 (UbcH5a) (Honda et al., 1997), while HOIP was used in combination with UBE2L3 (UbcH7) (Kirisako et al., 2006). In brief, the *in vitro* ubiquitylation reaction consisted of 1 mM ATP, 12.5, 6.25, and 3.125 μM ubiquitin, 50 nM E1, 250 nM E2, and 250 or 500 nM E3 ligase enzyme at 37°C for 30 min in a total volume of 5 μL (Figure 1A). Reactions were started by addition of ubiquitin and terminated by addition of 2.5 μL of 10% (v/v) trifluoroacetic acid. A dose of 1.05 μL of each reaction was then spiked with 300 nL (4 μM) of ^{15}N-labelled ubiquitin and 1.2 μL of 2,5-dihydroxyacetophenone matrix and 250 nL of this solution was spotted onto a 1,536 μL plate MALDI anchor target using a nanoliter dispensing robot. The samples were analyzed by high mass accuracy MALDI-TOF mass spectrometer (MS) in reflector positive ion mode on a rapifleX MALDI-TOF MS.

Importantly, the use of ^{15}N-labeled ubiquitin as internal standard allowed us not only to avoid spot-to-spot and shot-to-shot variability in MALDI ionization (Ritorto et al., 2014), but also it allowed us to keep track of the amount of mono-ubiquitin "consumed" during the assay. Overall, this setup allowed us to achieve very high precision, accuracy and reproducibility of measurements.

In our experimental conditions, ubiquitin consumption relied on the presence on an E3 ligase as we did not observe a significant reduction in the ubiquitin level within the negative controls (Figure 1B), where only E1 activating enzyme and E2 conjugating enzyme were present. Enzyme concentrations were optimized by reducing enzyme concentrations of previously reported SDS-PAGE auto-ubiquitylation assay protocols (Choo and Zhang, 2009; Zhao et al., 2012). An excess of ubiquitin (in the μM range) compared with the ubiquitin cascade enzymes

Figure 1. The MALDI-TOF E2/E3 Ligase Assay

(A) Workflow of the MALDI-TOF E2/E3 assay. Each of the three E3 ligases were incubated with their E2 partner with different concentrations of mono-ubiquitin (12.5, 6.25, and 3.125 μM) at 37°C. Reactions were stopped by addition of 2.5 μL 10% trifluoroacetic acid (TFA) at different time points. Reaction aliquots (1.05 μL) were mixed with 150 nL of 1.5 μM ^{15}N ubiquitin as internal standard. Subsequently, the analytes were mixed with **2,5-**dihydroxyacetophenone matrix and spotted onto a 1,536 AnchorChip MALDI target (Bruker Daltonics). Data analysis was performed using FlexAnalysis.

(B) E2/E3 ligase reactions are linear. Linearity is determined by mono-ubiquitin consumption over time. Only at very high and low concentrations of ubiquitin, the reaction is not linear. Data points have been normalized to determine reaction linearity. Data are represented as mean ± SD.

(C) Western blots of *in vitro* reactions (E1 100 nM, UBE2D1 250 nM, UBE2L3 125 nM, MDM2 and ITCH 500 nM, HOIP 250 nM, and ubiquitin 6.25 μM) of the three E3 ligases showing increased ubiquitin chain formation over time.

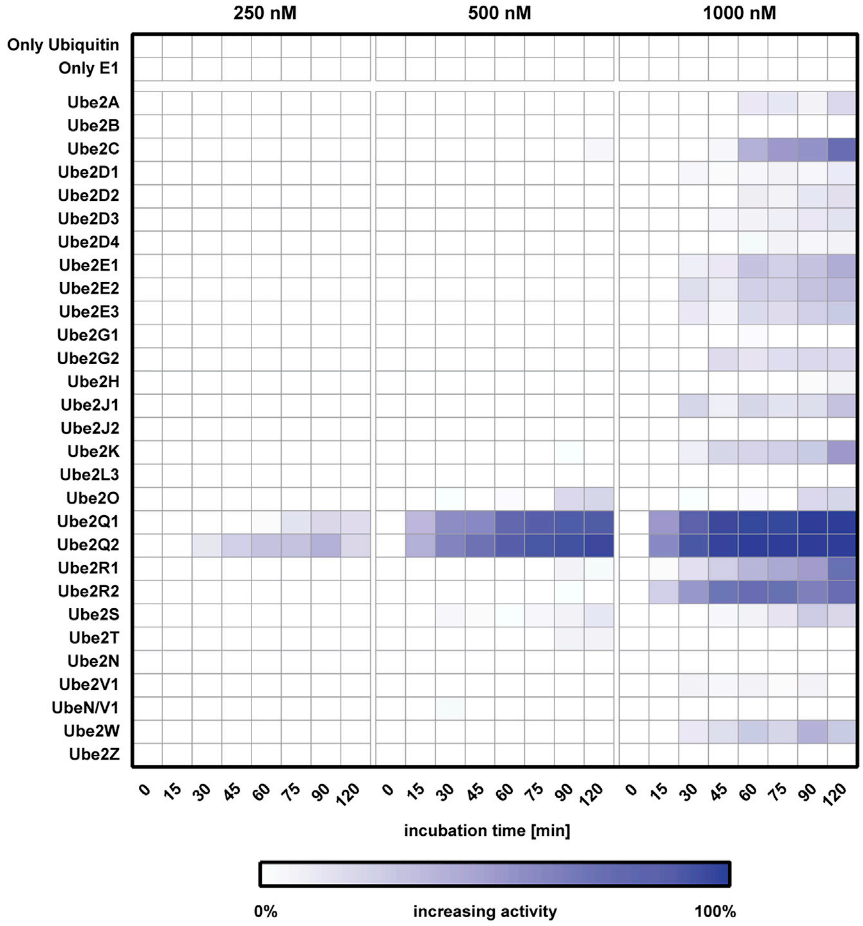

Figure 2. Characterizing E2 Enzyme *In Vitro* Activity

Twenty-seven E2 enzymes were incubated at three different concentrations (250, 500, and 1,000 nM) with the E1 UBE1 and 6.125 μM ubiquitin at 37°C. Reactions were stopped at the indicated time points with 2% TFA final and analyzed by MALDI-TOF mass spectrometry. Ligase activity was calculated considering T0 as 0% and the complete disappearance of mono-ubiquitin from the windows signal as 100% activity.

(250 nM for HOIP, 500 nM for ITCH, and 500 nM for MDM2) was found necessary in order to control reaction velocity. As expected, we observed that ubiquitin consumption was dose and enzyme dependent (Figure 1B, Figures S1 and S2). Reaction rates were related to ubiquitin concentration (Table S1) and different enzymes showed different rates of ubiquitin consumption (Figure 1B).

The well-established E2-E3 auto-ubiquitylation assays followed by SDS-PAGE and western blot analysis provided similar results, and we observed that the time-dependent disappearance of ubiquitin is comparable using both techniques (Figure 1C). Moreover, while substrate and enzyme concentrations are comparable with western blot-based approaches, the reaction volume (5 μL) is smaller than most of the antibody-based approaches currently reported in literature (Sheng et al., 2012).

Determining *In Vitro* Activities of E2 Enzymes

E2 ubiquitin conjugating enzymes are the central players in the ubiquitin cascade (Stewart et al., 2016). The human genome encodes ~40 E2 conjugating enzymes, of which about 30 conjugate ubiquitin directly while others conjugate small ubiquitin-like proteins such as SUMO1 and NEDD8 (Cappadocia and Lima, 2017). E2 enzymes are involved in every step of the ubiquitin chain formation pathway, from transferring the ubiquitin to mediating the switch from ubiquitin chain initiation to elongation, and defining the type of chain linkage. Connecting ubiquitin molecules in a defined manner by modifying specific Lys residues with ubiquitin is another intrinsic property of many E2 enzymes. Early studies showed that, at high concentrations, E2 enzymes can synthesize ubiquitin chains of a distinct linkage or undergo auto-ubiquitylation even in the absence of an E3 (Pickart and Rose, 1985), albeit at lower transfer rates (Stewart et al., 2016). This characteristic has been exploited for the generation of large amounts of different ubiquitin chain types *in vitro* (Faggiano et al., 2016).

As control for E2 enzyme mono or multi-ubiquitylation or E2-dependent ubiquitin chain assembly, we firstly assessed which E2 conjugating enzymes in our panel were able to consume ubiquitin even in absence of a partner E3 ligase. Utilizing the MALDI-TOF E2-E3 assay, we systematically tested 27 recombinantly expressed E2 conjugating enzymes (Table S2) for their ability to process ubiquitin either by the formation of polyubiquitin chains or by auto-ubiquitylation at different concentrations (250 nM, 500 nM, and 1 μM). We found that the UBE2Q1 and UBE2Q2 were able to consume ubiquitin even in absence of a specific E3 ligase at 250 nM after 45 min incubation time, and almost completely exhausting the starting ubiquitin amount after 2 hr of incubation (Figure 2). UBE2O and UBE2S are able to consume ubiquitin when present at a starting concentration of 500 nM, with consumption being evident from 90 min onward. Interestingly, UBE2Q1, UBE2Q2, and UBE2O (Berleth and Pickart, 1996; Klemperer et al., 1989; Melner et al., 2006) are E2 conjugating enzymes characterized by an unusually high molecular mass compared with other E2 enzymes: in particular, UBE2O has been reported as an E2-E3 hybrid which might explain its ability to form ubiquitin chains in the absence of an E3 ligase. Most of the E2 conjugating enzymes showed ubiquitin-consuming activity once their concentration was increased to 1 μM. Interestingly, UBE2D1 and UBE2L3 do not show any ligase activity, even at 1 μM, making these E2 conjugating enzymes the perfect candidates for inhibitor screening of E3 ligases as all ubiquitin-consuming activity in an assay will be down to E3 activity. Our results demonstrate that the E2-E3 MALDI-TOF assay has the potential to be employed for measuring E2 *in vitro* activity and therefore can be employed for screening inhibitors against

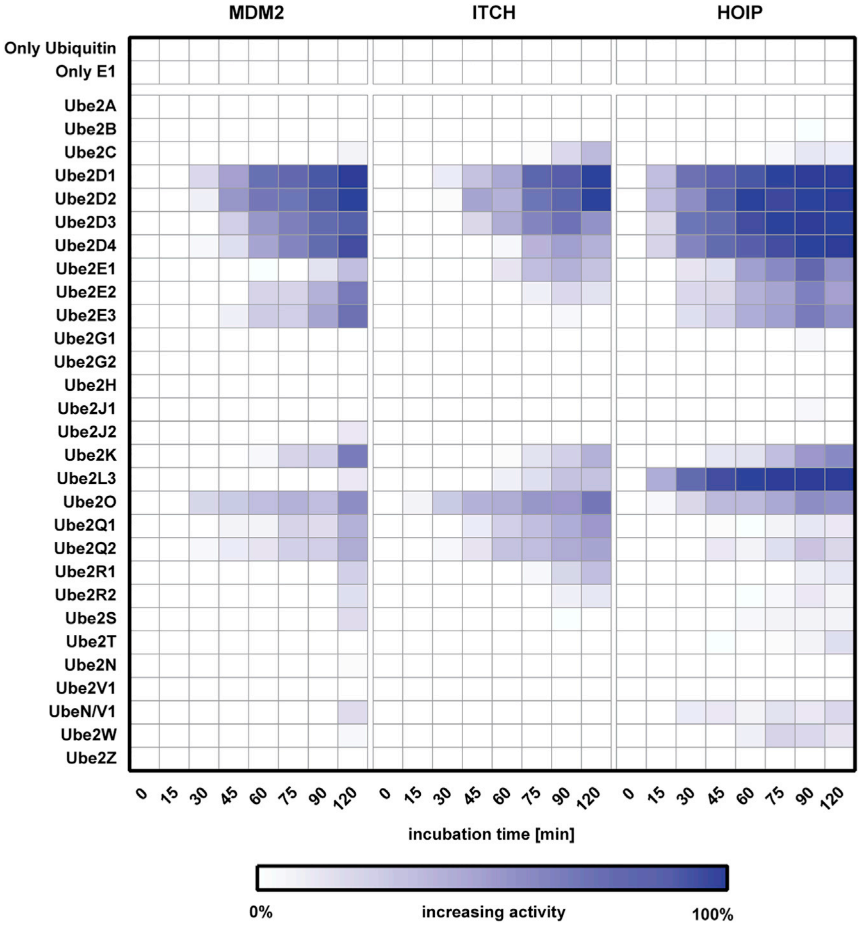

Figure 3. Characterizing E2/E3 Pair Activities

E1-E2 enzymes (50 and 250 nM, respectively) and E3 ligases were incubated in duplicate with 6.125 μM ubiquitin at 37°C for the time indicated. Reactions were stopped at the indicated time points with 2% TFA final and analyzed by MALDI-TOF mass spectrometry. Ligase activity was calculated considering T0 as 0% and the complete disappearance of mono-ubiquitin signal from the mass window as 100% activity.

those E2 enzymes that possess intrinsic ubiquitin ligase activity *in vitro*.

Determining E2/E3 Active Pairs

Any given E3 ligase cooperates with specific E2 enzymes *in vivo*. However, it is still difficult to predict which E2/E3 enzyme pair would be functional. Determining E2/E3 specificity is paramount to set up *in vitro* ubiquitylation assays and to perform inhibitor screens against E3 ligases. Using the E2/E3 MALDI-TOF assay, we investigated the activity of MDM2, ITCH, and HOIP throughout 8 time points when incubated with any of 27 ubiquitin E2 enzymes, covering the majority of the reported classes/families (Figure 3). We arbitrarily defined ‘‘fully active’’ pairs any E2-E3 couple that completely depleted the mono-ubiquitin starting amount after 2 hr incubation time. The UBE2D family is reported in the literature as being able to productively interact with MDM2 (Marblestone et al., 2013), ITCH (Sheng et al., 2012), and HOIP (Lechtenberg et al., 2016). Our data showed that UBE2D1 and UBE2D2, also known as UBCH5a and UBCH5b, were fully active with all the E3 ligases under investigation confirming the promiscuous activity of this class of E2 enzymes that was previously reported in the literature (Lechtenberg et al., 2016; Marblestone et al., 2013; Sheng et al., 2012). The UBE2E family was only partially active against the E3s ligases of interest. The well-characterized human E2L3 (or UBCH7) showed activity with HOIP and ITCH, confirming the already reported UBCH7 ability of functioning with both HECT and RBR E3 families (Capili et al., 2004; Shimura et al., 2000). Taken together these results demonstrate that the E2/E3 MALDI-TOF assay is suitable for determining E2 specificity toward their cognate E3 enzymes in a high-throughput fashion.

Assessing Potency and Selectivity of E2/E3 Inhibitors

We next evaluated whether the MALDI-TOF E2/E3 assay had the potential to assess the potency and selectivity of E2/E3 inhibitors. As proof-of-concept, we tested five inhibitors that had previously been reported to inhibit E1, E2, or E3 ligases: PYR41 (Yang et al., 2007), BAY117082 (Strickson et al., 2013), gliotoxin (Sakamoto et al., 2015), nutlin-3A (Vassilev et al., 2004), clomipramine (Rossi et al., 2014), and Compound 1 (Brownell et al., 2010). We also tested PR619 (Altun et al., 2011), a broad-spectrum, alkylating deubiquitylase (DUB) inhibitor, which we hypothesized would also inhibit other enzymes with active site cysteines, such as E1/E2 enzymes and E3 ligases. PYR41 is a specific and cell-permeable inhibitor of E1 ubiquitin loading but does not directly affect E2 activity (Yang et al., 2007). BAY117082, initially described as inhibitor of NF-κB phosphorylation, has been shown to inactivate the E2 conjugating enzymes Ubc13 (UBE2N) and UBCH7 (UBE2L3), as well as the E3 ligase LUBAC (of which HOIP is part) (Strickson et al., 2013). Gliotoxin is a fungal metabolite identified as a selective inhibitor of HOIP through an fluorescence resonance energy transfer (FRET)-based HTS assay (Sakamoto et al., 2015). Nutlin 3A is a MDM2-p53 interaction inhibitor, able to displace p53 from MDM2 with an IC_{50} in the 100–300 nM range (Khoury and Domling, 2012). However nutlins are not reported to be able to inhibit MDM2 auto-ubiquitylation. Compound 1 is a pan-inhibitor of ubiquitin-like activating enzymes, an analog of the NEDD8 activating enzyme inhibitor MLN4924 (Rossi et al., 2014; Soucy et al., 2009). It forms a covalent adduct with the ubiquitin-like substrate through its sulfamate group in a process that requires Mg-ATP. Clomipramine is a compound reported as able to block ITCH ubiquitin transthiolation in an irreversible manner, achieving complete inhibition at 0.8 mM (Rossi et al., 2014). However, in our hands it did not inhibit any of the E3 ligases at

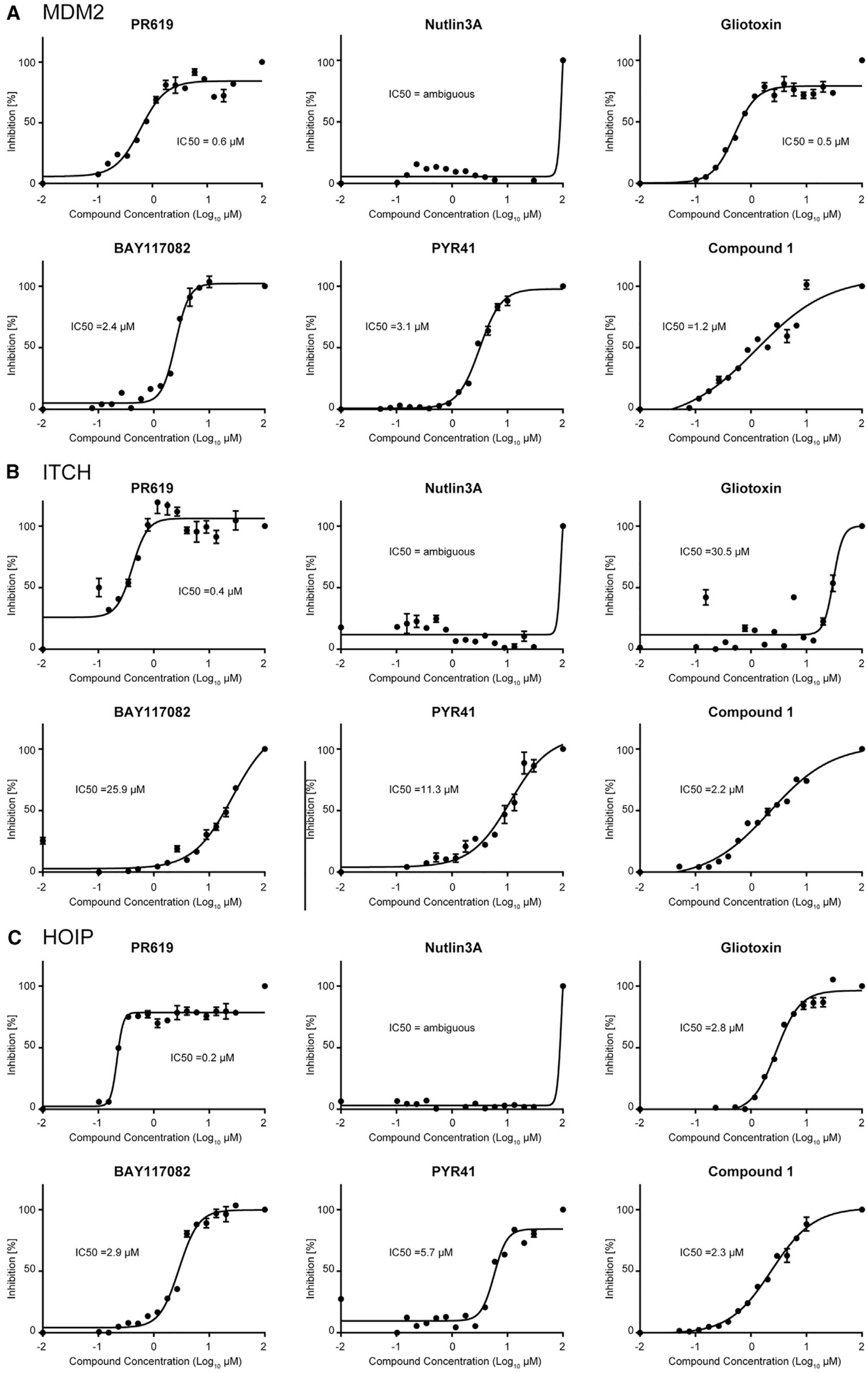

(legend on next page)

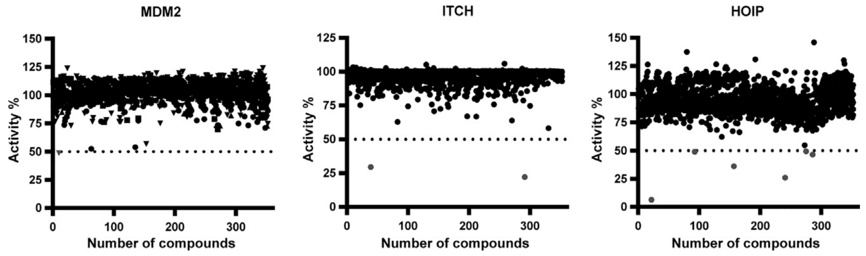

Figure 5. High-Throughput E2/E3 MALDI-TOF Screen

A total of 1,430 compounds from various commercial suppliers were tested at a final concentration of 10 μM each against MDM2, ITCH, and HOIP. The uninhibited control contained 5 nL DMSO but no compound, whereas the inhibited control had been inactivated by pre-treatment with 2.0% TFA.

10 μM, and we determined its IC_{50} for ITCH to be ~500 μM (Figure S3). We therefore did not follow up clomipramine.

Performing IC_{50} inhibition curves using the MALDI-TOF E2/E3 assay (Figure 4; Table S3), we could show that PYR41 inhibited MDM2, ITCH, and HOIP at IC_{50} values of 3.1, 11.3, and 5.7 μM. BAY117082 also strongly inhibited MDM2 with an IC_{50} value of 2.4 μM, and HOIP and ITCH with IC_{50} values of 2.9 and 25.9 μM, respectively. Gliotoxin showed an IC_{50} value of 2.8 μM against HOIP, of 30.5 μM against ITCH, and 0.5 μM against MDM2. As expected, nutlin-3A did not show any inhibitory activity toward MDM2, ITCH, or HOIP, as it was designed as an interaction inhibitor. We found that Compound 1 inhibited the reactions with all three E3 ligases with similar potencies at 1–2 μM, probably by inhibiting the E1 enzyme. PR619 resulted as the most powerful inhibitor of the ubiquitination cascade, with an IC_{50} of 0.6 μM against MDM2, 0.4 μM against ITCH, and 0.2 μM when tested against HOIP, suggesting that PR619, which also acts as a DUB inhibitor (Ritorto et al., 2014), has a very low degree of selectivity and inhibits many enzymes with active cysteines.

To test if these compounds can covalently modify E2 enzymes, we used MALDI-TOF MS of the intact proteins incubated with the inhibitor compounds. We found that BAY117082 and PR619 both covalently modified E2 enzymes (Figure S4). BAY117082 has been previously characterized for its ability to bind to a cysteine of UBE2N resulting in a mass shift of 51 Da (Strickson et al., 2013). Here we observed that BAY117082 covalently bound to UBE2L3 resulting in a mass shift of 153 Da, corresponding to the addition of three molecules of BAY117082 to the three cysteines encoded within its sequence. The covalent binding of multiple BAY117082 and PR619 molecules to the E2 might explain the high values for the Hill coefficients (see Table S4) when performing IC_{50} curves. We also would like to point out that we noticed that the initial presence of β-mercaptoethanol (BME) in the MDM2 preparation suppressed the inhibition effect of PYR41 and BAY117082. Removal of BME showed the inhibition as presented in Figure 4. We therefore recommend avoiding thiol-based reducing agents such as BME or DTT when testing compounds that target active cysteines which are present in most of the ubiquitin pathway enzymes. Both of these reducing agents can be replaced with (tris(2-carboxyethyl)phosphine.

Overall, our results demonstrate that the MALDI-TOF E2-E3 assay is suitable for comparing inhibitor potency through IC_{50} determination.

E2/E3 Assay by MALDI-TOF Is Suitable for HTS

Having established that the E2/E3 MALDI-TOF assay can be used to assess the specificity and potency of inhibitors, we explored its suitability for HTS. It is important to underline that, because of the nature of the assay, inhibitors of E1 or E2, which both contain active site cysteines (Rossi et al., 2014; Sakamoto et al., 2015) may be identified as hits. We tested a library of 1,430 FDA-approved compounds from various commercial suppliers with validated biological and pharmacological activities at 10 μM final. None of the compounds present in the library are known for specifically targeting MDM2, ITCH, or HOIP. The assay was performed supplying ATP in excess (1 mM) to reduce the likelihood of identifying ATP analogs as inhibitors of these enzymes.

The screens against the three different E3 ligases, expressed as percentage effect (Figures 5 and S5) exhibited robust Z' scores >0.5 (Table S5). These scores provide a measure for the suitability of screening assays; HTS assays that provide Z' scores >0.5 are generally considered robust.

We defined as a positive hit a compound whose potency ranked above the 50% residual activity threshold. Overall, we identified nine compounds reporting inhibition rates >50% against the E3 ligases of interest. Candesartan cilexetil was the only compound able to inhibit MDM2 activity by more than 50% (see Table 1). With regard to HOIP screening, six compounds were identified as potential inhibitors: bendamustine, moclobemide, ebselen, cefatrizine, fluconazole, and pyrazinamide. The ITCH inhibitor screening identified two positive hits: hexachlorophene and ethacrynic acid. Hexachlorophene is an organochloride compound once widely used as a disinfectant. It acts as an alkylating agent, thus resulting in the wide and not specific inhibition of E3 ligases: this explains why this compound results as a weak inhibitor of MDM2 as well. Ethacrynic acid is a diuretic compound (Melvin et al., 1963) and a potent inhibitor of glutathione S-transferase, with intrinsic chemical reactivity toward sulfhydryl groups (Koechel, 1981), which might explains its ranking as positive hit in our assay when tested against both MDM2 and ITCH. Overall,

Figure 4. IC_{50} Analyses of six inhibitors for Selected E3 Ligases

IC_{50} determination for six described E3 ligase inhibitors for MDM2 (A), ITCH (B), and HOIP (C). Small inhibitor compounds were pre-incubated for 30 min at different concentrations (0–100 μM). Ubiquitin was added and incubated for a range of time depending on the E3 (usually 30 or 40 min). For statistical analysis, Prism GraphPad software was used with a built-in analysis, nonlinear regression (curve-fit), variable slope (four parameters) curve to determine IC_{50} values. Data are represented as mean ± SD.

Table 1. Positive Hits Identified by E2/E3 MALDI-TOF Assay

E2/E3 Ligase	Compound Name	Residual Activity (%)
UBE1/UBE2L3/ HOIP	bendamustine	6.3
	moclobemide	25.9
	ebselen	36.2
	fluconazole	46.4
	cefatrizine	49.0
	pyrazinamide	49.3
	nevirapine	54.8
	resveratrol	62.2
	bupivacaine	66.2
	2-(3,4-dimethoxyphenyl) ethanamine	68.2
UBE1/UBE2D1/ ITCH	hexachlorophene	22.2
	ethacrynic acid	29.5
	alosetron	58.2
	vecuronium or pancuronium	67
UBE1/UBE2D1/ MDM2	candesartan cilexetil	48.6
	hexachlorophene	52.4
	ethacrynic acid	53.9
	aztreonam	56.7

our results demonstrate that the E2/E3 MALDI-TOF assay can be employed to screen large compound libraries against E1, E2 conjugating enzymes and E3 ligases belonging to different families for the identification of new inhibitors in the ubiquitin pathway.

Validation of Positive Hits

To validate the results obtained from the HTS we performed IC_{50} determination of compounds with the highest inhibitor potency in the single point screening. Candesartan cilexitel, an angiotensin II receptor antagonist, inhibited MDM2 with an IC_{50} of 8.8 μM (Figure 6A). Best hit was bendamustine, a nitrogen mustard used in the treatment of chronic lymphocytic leukemia and lymphomas. It belongs to the family of alkylating agents. Bendamustine ranked as the compound with the highest inhibition score against HOIP, while it did not significantly affect MDM2 and ITCH activities. We confirmed that bendamustine selectively inhibited HOIP at an IC_{50} of 6.4 μM, while ITCH and MDM2 showed a considerably higher IC_{50} value of 76.8 μM and 114 μM, respectively (Figure 6B). Bendamustine retained its inhibition power when HOIP was paired with UBE2D1 as conjugating enzyme (Figure S6), suggesting that the compound binds preferentially to HOIP. This shows that the MALDI-TOF E2/E3 ligase assay can be used to identify selective inhibitors from a high-throughput screen.

DISCUSSION

The ubiquitin system has in recent years become an exciting area for drug discovery (Cohen and Tcherpakov, 2010), as multiple enzymatic steps within the ubiquitylation process are druggable. The potential of targeting the ubiquitin-proteasome pathway was first demonstrated in 2003 by the approval of the proteasome inhibitor bortezomib (Velcade; Millennium Pharmaceuticals) for use in multiple myeloma. While proteasome inhibition is a broad intervention affecting general survivability, E3 ubiquitin ligases and DUBs (Ritorto et al., 2014) represent the most specific points of intervention for therapeutic tools as they specifically regulate the ubiquitylation rate of specific substrates. For example, nutlins, *cis*-imidazoline analogs able to inhibit the interaction between MDM2 and tumor suppressor p53, have recently entered early clinical trials for the treatment of blood cancers (Burgess et al., 2016). The small number of drugs targeting E3 ligases currently on the market is partly due to the lack of suitable high-throughput assays for drug discovery screening. Traditionally, screening for inhibitors of ubiquitin ligases and DUBs has been performed using different fluorescence-based formats in high-throughput and ELISA, SDS-PAGE, and western blotting in low-throughput. These approaches show a number of limitations. ELISA- and SDS-PAGE-based approaches are time consuming and low-throughput by nature, and therefore mostly incompatible with HTS. The applicability of fluorescence-based techniques such as FRET is dependent on being able to get FRET donors and acceptors in the right distance, and the fluorescent label might affect inhibitor binding. To address these issues, we have developed a sensitive and fast assay to quantify *in vitro* E2/E3 enzyme activity using MALDI-TOF MS. It builds on our DUB MALDI-TOF assay (Ritorto et al., 2014), which has enabled us to screen successfully for a number of selective DUB inhibitors (Kategaya et al., 2017; Magiera et al., 2017; Weisberg et al., 2017), and adds to the increasing number of drug discovery assays utilizing label-free high-throughput MALDI-TOF MS. Apart from E2/E3 enzymes and DUBs (Ritorto et al., 2014), high-throughput MALDI-TOF MS has now successfully been used for drug discovery screening of protein kinases (Heap et al., 2017b), protein phosphatases (Winter et al., 2018), histone demethylases, and acetylcholinesterases (Haslam et al., 2016), as well as histone lysine methyltransferases (Guitot et al., 2014).

Unlike other current assays, all these label-free MALDI-TOF MS methods use unmodified substrates, such as mono-ubiquitin. The advantages compared with fluorescence or antibody-based high-throughput assays is the ability to work with enzymes without the previous development of specific chemical/fluorescent probes, as well as the reduced consumable costs for the assay as no antibodies are required. Moreover, because of the sensitivity of current MALDI-TOF MSs, all enzymes are usually kept at low concentrations thereby significantly reducing the amounts and cost per assay.

In the context of E3 ligase drug discovery, it is critical to identify the appropriate E2/E3 substrate pairing to ensure the development and use of the most physiologically relevant screening assay. There have been many reports of limited E2/E3 activity profiling with a small number of E2 and E3 enzymes using ELISA-based assays, structural-based yeast two-hybrid assays, and western blot (Lechtenberg et al., 2016; Marblestone et al., 2013; Sheng et al., 2012). All of these approaches are time consuming, require large amounts of reagents, and are difficult to adapt for HTS. We have successfully used our E2/E3 MALDI-TOF assay to identify active E2/E3 pairings, which

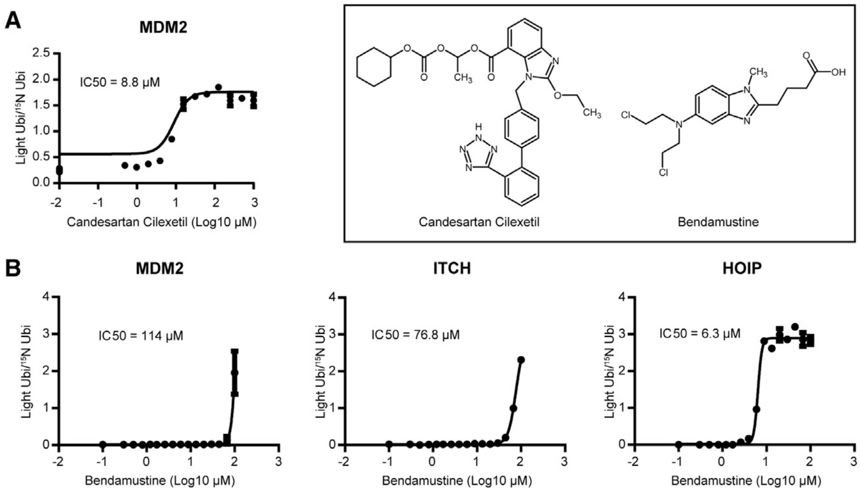

Figure 6. Validation of Hits

(A) Candesartan Cilexetil shows an IC_{50} of 8.8 μM against MDM2.

(B) Bendamustine shows some specificity for HOIP (IC_{50} = 6.3 μM) while inhibiting MDM2 (IC_{50} = 114 μM) and ITCH (IC_{50} = 76.8 μM) at higher concentrations. Data are represented as mean ± SD.

could then be further characterized using our HTS screen. The "E2 scan" was quickly and easily adapted, collecting data of three E3 enzymes against 29 E2 enzymes at 8 time points in one single experiment. Moreover, after identification of the right E2/E3 pairs, we applied the MALDI-TOF E2/E3 assay to determine inhibition rates and the IC_{50} values of small-molecule inhibitors. In a proof-of-concept study, we performed an HTS for inhibitors of three E3 ligases. The MALDI-TOF analysis speed of 1.3 s per sample (~35 min per 1,536-well plate) and low sample volumes (reaction volume 5 μL/MALDI deposition 250 nL) make the E2/E3 MALDI-TOF assay comparable with other fluorescence/chemical probe-based technologies. Automatic sample preparation, MALDI-TOF plate spotting, and data collection allowed us to quickly analyze thousands of compounds through the use of 1,536 sample targets. The assay successfully identified bendamustine as a novel small-molecule inhibitor for HOIP, an attractive drug target for both inflammatory disease and cancer (Ikeda et al., 2011; MacKay et al., 2014; McGuire et al., 2016; Stieglitz et al., 2013). Bendamustine, a nitrogen mustard, shows likely very high reactivity against a range of targets in the cell including its intended target DNA. However, it is surprising that it shows a 12- and 18-fold higher activity against HOIP than against ITCH and MDM2, respectively, suggesting that there is possibly a structural effect and some selectivity can be reached between different E3 ligases. It also shows that E1 conjugating enzymes were not affected by bendamustine as the same enzyme was also used in the MDM and ITCH reaction. While this is just a proof-of-concept study characterizing E2/E3 activity and identifying inhibitors in an *in vitro* system, follow-up studies will need to verify results in cellular and ultimately *in vivo* models.

In conclusion, we present here a novel screening method to assay E2/E3 activity with high sensitivity, reproducibility, and reliability, which is able to carry out precise quantified measurements at a rate of ~1 s per sample spot. Using physiological substrates, we showed proof-of concept for three E3 ligases that are attractive drug targets. Considering the speed, low consumable costs, and the simplicity of the assay, the MALDI-TOF E3 ligase assay will serve as a sensitive and fast tool for screening for E1, E2 enzyme, and E3 ligase inhibitors.

SIGNIFICANCE

Our understanding of the ubiquitin biology has been rapidly expanding. The role of the ubiquitin system in the pathogenesis of numerous disease states has increased the interest in finding new strategies to pharmacologically interfere with the enzymes responsible of the ubiquitination process. However, the development of molecules targeting the ubiquitin cascade, especially the E2 conjugating enzymes and E3 ligases, has not being extensively sustained by the availability of robust and affordable technologies for extensive primary screening of compound libraries. Performing high-throughput screening in the ubiquitin field remains challenging and it usually requires engineered proteins or the synthesis of chemical probes. Here we show that the MALDI-TOF E2-E3 assay is a robust, scalable, label-free assay that can be employed for primary screening of compound libraries against E2 conjugating enzymes and E3 ligases belonging to different families and representative of all the currently known ubiquitylation mechanisms. The MALDI-TOF E2/E3 assay is a readily accessible addition to the drug discovery toolbox with the potential to accelerate drug discovery efforts in the ubiquitin pathway.

STAR★METHODS

Detailed methods are provided in the online version of this paper and include the following:

- KEY RESOURCES TABLE
- CONTACT FOR REAGENT AND RESOURCE SHARING
- EXPERIMENTAL MODEL AND SUBJECT DETAILS
  - E1, E2, ITCH and HOIP E3 Enzyme Expression and Purification
  - MDM2 E3 Enzyme Expression and Purification
- METHOD DETAILS
  - E1/E2/E3 Assay
  - Compound Library Spotting and Inhibitor Screening
  - Target Spotting and MALDI Mass Spectrometry Analysis
  - Data Analysis
- QUANTIFICATION AND STATISTICAL ANALYSIS

SUPPLEMENTAL INFORMATION

Supplemental Information includes six figures and five tables and can be found with this article online at https://doi.org/10.1016/j.chembiol.2018.06.004.

ACKNOWLEDGMENTS

We would like to thank the DNA cloning, Protein Production, DNA sequencing facility, and mass spectrometry teams of the MRC Protein Phosphorylation and Ubiquitylation Unit for their support. We would like to thank Prof. Dario Alessi, Prof. Ronald Hay, Prof. Philip Cohen, Prof. Katrin Rittinger, Dr. Satpal Virdee, Dr. Sarah Buhrlage, Dr. Natalia Shpiro, Dr. Siddharth Bakshi, Dr. Andrea Testa, and Dr. Francesca Morreale for tools and helpful discussions; Bruker Daltonics, particularly Meike Hamester, Rainer Paape, and Anja Resemann for their technical support. We thank Dr. Anthony Hope, Alex Cookson, and Lorna Campbell for providing the FDA-approved compound library and support with the liquid handling. This work was funded by Medical Research Council UK (MC_UU_12016/5), and the pharmaceutical companies supporting the Division of Signal Transduction Therapy (DSTT) (Boehringer-Ingelheim, GlaxoSmithKline, and Merck KGaA).

AUTHOR CONTRIBUTIONS

V.D.C. performed all experiments. C.J., V.B., A.K., and J.H. produced the proteins. V.D.C. and M.T. designed the experiments. V.D.C. and M.T. prepared the manuscript with contributions from all authors.

DECLARATION OF INTERESTS

The authors have no financial interests to declare.

Received: December 8, 2017
Revised: April 14, 2018
Accepted: June 8, 2018
Published: July 12, 2018

REFERENCES

Altun, M., Kramer, H.B., Willems, L.I., McDermott, J.L., Leach, C.A., Goldenberg, S.J., Kumar, K.G., Konietzny, R., Fischer, R., Kogan, E., et al. (2011). Activity-based chemical proteomics accelerates inhibitor development for deubiquitylating enzymes. Chem. Biol. *18*, 1401–1412.

Berleth, E.S., and Pickart, C.M. (1996). Mechanism of ubiquitin conjugating enzyme E2-230K: catalysis involving a thiol relay? Biochemistry *35*, 1664–1671.

Brownell, J.E., Sintchak, M.D., Gavin, J.M., Liao, H., Bruzzese, F.J., Bump, N.J., Soucy, T.A., Milhollen, M.A., Yang, X., Burkhardt, A.L., et al. (2010). Substrate-assisted inhibition of ubiquitin-like protein-activating enzymes: the NEDD8 E1 inhibitor MLN4924 forms a NEDD8-AMP mimetic in situ. Mol. Cell *37*, 102–111.

Burgess, A., Chia, K.M., Haupt, S., Thomas, D., Haupt, Y., and Lim, E. (2016). Clinical overview of MDM2/X-targeted therapies. Front. Oncol. *6*, 7.

Capili, A.D., Edghill, E.L., Wu, K., and Borden, K.L. (2004). Structure of the C-terminal RING finger from a RING-IBR-RING/TRIAD motif reveals a novel zinc-binding domain distinct from a RING. J. Mol. Biol. *340*, 1117–1129.

Cappadocia, L., and Lima, C.D. (2017). Ubiquitin-like protein conjugation: structures, chemistry, and mechanism. Chem. Rev. *118*, 889–918.

Chaugule, V.K., and Walden, H. (2016). Specificity and disease in the ubiquitin system. Biochem. Soc. Trans. *44*, 212–227.

Choo, Y.S., and Zhang, Z. (2009). Detection of protein ubiquitination. J. Vis. Exp. https://doi.org/10.3791/1293.

Cohen, P., and Tcherpakov, M. (2010). Will the ubiquitin system furnish as many drug targets as protein kinases? Cell *143*, 686–693.

Davydov, I.V., Woods, D., Safiran, Y.J., Oberoi, P., Fearnhead, H.O., Fang, S., Jensen, J.P., Weissman, A.M., Kenten, J.H., and Vousden, K.H. (2004). Assay for ubiquitin ligase activity: high-throughput screen for inhibitors of HDM2. J. Biomol. Screen. *9*, 695–703.

de Bie, P., and Ciechanover, A. (2011). Ubiquitination of E3 ligases: self-regulation of the ubiquitin system via proteolytic and non-proteolytic mechanisms. Cell Death Differ. *18*, 1393–1402.

Dudgeon, D.D., Shinde, S., Hua, Y., Shun, T.Y., Lazo, J.S., Strock, C.J., Giuliano, K.A., Taylor, D.L., Johnston, P.A., and Johnston, P.A. (2010). Implementation of a 220,000-compound HCS campaign to identify disruptors of the interaction between p53 and hDM2 and characterization of the confirmed hits. J. Biomol. Screen. *15*, 766–782.

Faggiano, S., Alfano, C., and Pastore, A. (2016). The missing links to link ubiquitin: methods for the enzymatic production of polyubiquitin chains. Anal Biochem. *492*, 82–90.

Fakharzadeh, S.S., Trusko, S.P., and George, D.L. (1991). Tumorigenic potential associated with enhanced expression of a gene that is amplified in a mouse tumor cell line. EMBO J. *10*, 1565–1569.

Guitot, K., Scarabelli, S., Drujon, T., Bolbach, G., Amoura, M., Burlina, F., Jeltsch, A., Sagan, S., and Guianvarc'h, D. (2014). Label-free measurement of histone lysine methyltransferases activity by matrix-assisted laser desorption/ionization time-of-flight mass spectrometry. Anal Biochem. *456*, 25–31.

Hansen, T.M., Rossi, M., Roperch, J.P., Ansell, K., Simpson, K., Taylor, D., Mathon, N., Knight, R.A., and Melino, G. (2007). Itch inhibition regulates chemosensitivity in vitro. Biochem. Biophys. Res. Commun. *361*, 33–36.

Haslam, C., Hellicar, J., Dunn, A., Fuetterer, A., Hardy, N., Marshall, P., Paape, R., Pemberton, M., Resemannand, A., and Leveridge, M. (2016). The evolution of MALDI-TOF mass spectrometry toward ultra-high-throughput screening: 1536-well format and beyond. J. Biomol. Screen. *21*, 176–186.

Hatakeyama, S. (2011). TRIM proteins and cancer. Nat. Rev. Cancer *11*, 792–804.

Heap, R.E., Gant, M.S., Lamoliatte, F., Peltier, J., and Trost, M. (2017a). Mass spectrometry techniques for studying the ubiquitin system. Biochem. Soc. Trans. *45*, 1137–1148.

Heap, R.E., Hope, A.G., Pearson, L.A., Reyskens, K., McElroy, S.P., Hastie, C.J., Porter, D.W., Arthur, J.S.C., Gray, D.W., and Trost, M. (2017b). Identifying inhibitors of inflammation: a novel high-throughput MALDI-TOF screening assay for salt-inducible kinases (SIKs). SLAS Discov. *22*, 1193–1202.

Honda, R., Tanaka, H., and Yasuda, H. (1997). Oncoprotein MDM2 is a ubiquitin ligase E3 for tumor suppressor p53. FEBS Lett. *420*, 25–27.

Huang, J., Sheung, J., Dong, G., Coquilla, C., Daniel-Issakani, S., and Payan, D.G. (2005). High-throughput screening for inhibitors of the e3 ubiquitin ligase APC. Methods Enzymol. *399*, 740–754.

Ikeda, F., Deribe, Y.L., Skanland, S.S., Stieglitz, B., Grabbe, C., Franz-Wachtel, M., van Wijk, S.J., Goswami, P., Nagy, V., Terzic, J., et al. (2011). SHARPIN forms a linear ubiquitin ligase complex regulating NF-kappaB activity and apoptosis. Nature *471*, 637–641.

Kategaya, L., Di Lello, P., Rouge, L., Pastor, R., Clark, K.R., Drummond, J., Kleinheinz, T., Lin, E., Upton, J.P., Prakash, S., et al. (2017). USP7 small-molecule inhibitors interfere with ubiquitin binding. Nature *550*, 534–538.

Kenten, J.H., Davydov, I.V., Safiran, Y.J., Stewart, D.H., Oberoi, P., and Biebuyck, H.A. (2005). Assays for high-throughput screening of E2 AND E3 ubiquitin ligases. Methods Enzymol. *399*, 682–701.

Khoury, K., and Domling, A. (2012). P53 mdm2 inhibitors. Curr. Pharm. Des. *18*, 4668–4678.

Kim, W., Bennett, E.J., Huttlin, E.L., Guo, A., Li, J., Possemato, A., Sowa, M.E., Rad, R., Rush, J., Comb, M.J., et al. (2011). Systematic and quantitative assessment of the ubiquitin-modified proteome. Mol. Cell *44*, 325–340.

Kirisako, T., Kamei, K., Murata, S., Kato, M., Fukumoto, H., Kanie, M., Sano, S., Tokunaga, F., Tanaka, K., and Iwai, K. (2006). A ubiquitin ligase complex assembles linear polyubiquitin chains. EMBO J. *25*, 4877–4887.

Klemperer, N.S., Berleth, E.S., and Pickart, C.M. (1989). A novel, arsenite-sensitive E2 of the ubiquitin pathway: purification and properties. Biochemistry *28*, 6035–6041.

Koechel, D.A. (1981). Ethacrynic acid and related diuretics: relationship of structure to beneficial and detrimental actions. Annu. Rev. Pharmacol. Toxicol. *21*, 265–293.

Krist, D.T., Park, S., Boneh, G.H., Rice, S.E., and Statsyuk, A.V. (2016). UbFluor: a mechanism-based probe for HECT E3 ligases. Chem. Sci. *7*, 5587–5595.

Lechtenberg, B.C., Rajput, A., Sanishvili, R., Dobaczewska, M.K., Ware, C.F., Mace, P.D., and Riedl, S.J. (2016). Structure of a HOIP/E2∼ubiquitin complex reveals RBR E3 ligase mechanism and regulation. Nature *529*, 546–550.

Levin-Kravets, O., Tanner, N., Shohat, N., Attali, I., Keren-Kaplan, T., Shusterman, A., Artzi, S., Varvak, A., Reshef, Y., Shi, X., et al. (2016). A bacterial genetic selection system for ubiquitylation cascade discovery. Nat. Methods *13*, 945–952.

Lorick, K.L., Jensen, J.P., Fang, S., Ong, A.M., Hatakeyama, S., and Weissman, A.M. (1999). RING fingers mediate ubiquitin-conjugating enzyme (E2)-dependent ubiquitination. Proc. Natl. Acad. Sci. USA *96*, 11364–11369.

MacKay, C., Carroll, E., Ibrahim, A.F.M., Garg, A., Inman, G.J., Hay, R.T., and Alpi, A.F. (2014). E3 ubiquitin ligase HOIP attenuates apoptotic cell death induced by cisplatin. Cancer Res. *74*, 2246–2257.

Maculins, T., Carter, N., Dorval, T., Hudson, K., Nissink, J.W., Hay, R.T., and Alwan, H. (2016). A generic platform for cellular screening against ubiquitin ligases. Sci. Rep. *6*, 18940.

Magiera, K., Tomala, M., Kubica, K., De Cesare, V., Trost, M., Zieba, B.J., Kachamakova-Trojanowska, N., Les, M., Dubin, G., Holak, T.A., et al. (2017). Lithocholic acid hydroxyamide destabilizes cyclin D1 and induces G0/G1 arrest by inhibiting deubiquitinase USP2a. Cell Chem. Biol. *24*, 458–470 e418.

Marblestone, J.G., Butt, S., McKelvey, D.M., Sterner, D.E., Mattern, M.R., Nicholson, B., and Eddins, M.J. (2013). Comprehensive ubiquitin E2 profiling of ten ubiquitin E3 ligases. Cell Biochem. Biophys. *67*, 161–167.

Marblestone, J.G., Larocque, J.P., Mattern, M.R., and Leach, C.A. (2012). Analysis of ubiquitin E3 ligase activity using selective polyubiquitin binding proteins. Biochim. Biophys. Acta *1823*, 2094–2097.

Marblestone, J.G., Suresh Kumar, K.G., Eddins, M.J., Leach, C.A., Sterner, D.E., Mattern, M.R., and Nicholson, B. (2010). Novel approach for characterizing ubiquitin E3 ligase function. J. Biomol. Screen. *15*, 1220–1228.

McGuire, V.A., Ruiz-Zorrilla Diez, T., Emmerich, C.H., Strickson, S., Ritorto, M.S., Sutavani, R.V., Weibeta, A., Houslay, K.F., Knebel, A., Meakin, P.J., et al. (2016). Dimethyl fumarate blocks pro-inflammatory cytokine production via inhibition of TLR induced M1 and K63 ubiquitin chain formation. Sci. Rep. *6*, 31159.

Melner, M.H., Haas, A.L., Klein, J.M., Brash, A.R., Boeglin, W.E., Nagdas, S.K., Winfrey, V.P., and Olson, G.E. (2006). Demonstration of ubiquitin thiolester formation of UBE2Q2 (UBCi), a novel ubiquitin-conjugating enzyme with implantation site-specific expression. Biol. Reprod. *75*, 395–406.

Melvin, K.E., Farrelly, R.O., and North, J.D. (1963). Ethacrynic acid: a new oral diuretic. Br. Med. J. *1*, 1521–1524.

Pickart, C.M. (2001). Mechanisms underlying ubiquitination. Annu. Rev. Biochem. *70*, 503–533.

Pickart, C.M., and Rose, I.A. (1985). Functional heterogeneity of ubiquitin carrier proteins. J. Biol. Chem. *260*, 1573–1581.

Ranaweera, R.S., and Yang, X. (2013). Auto-ubiquitination of Mdm2 enhances its substrate ubiquitin ligase activity. J. Biol. Chem. *288*, 18939–18946.

Regnstrom, K., Yan, J., Nguyen, L., Callaway, K., Yang, Y., Diep, L., Xing, W., Adhikari, A., Beroza, P., Hom, R.K., et al. (2013). Label free fragment screening using surface plasmon resonance as a tool for fragment finding - analyzing parkin, a difficult CNS target. PLoS One *8*, e66879.

Ritorto, M.S., Ewan, R., Perez-Oliva, A.B., Knebel, A., Buhrlage, S.J., Wightman, M., Kelly, S.M., Wood, N.T., Virdee, S., Gray, N.S., et al. (2014). Screening of DUB activity and specificity by MALDI-TOF mass spectrometry. Nat. Commun. *5*, 4763.

Rivetti di Val Cervo, P., Tucci, P., Majid, A., Lena, A.M., Agostini, M., Bernardini, S., Candi, E., Cohen, G., Nicotera, P., Dyer, M.J., et al. (2009). p73, miR106b, miR34a, and Itch in chronic lymphocytic leukemia. Blood *113*, 6498–6499, author reply 6499–6500.

Rivlin, N., Brosh, R., Oren, M., and Rotter, V. (2011). Mutations in the p53 tumor suppressor gene: important milestones at the various steps of tumorigenesis. Genes Cancer *2*, 466–474.

Rossi, M., Inoue, S., Walewska, R., Knight, R.A., Dyer, M.J., Cohen, G.M., and Melino, G. (2009). Caspase cleavage of Itch in chronic lymphocytic leukemia cells. Biochem. Biophys. Res. Commun. *379*, 659–664.

Rossi, M., Rotblat, B., Ansell, K., Amelio, I., Caraglia, M., Misso, G., Bernassola, F., Cavasotto, C.N., Knight, R.A., Ciechanover, A., et al. (2014). High throughput screening for inhibitors of the HECT ubiquitin E3 ligase ITCH identifies antidepressant drugs as regulators of autophagy. Cell Death Dis. *5*, e1203.

Sakamoto, H., Egashira, S., Saito, N., Kirisako, T., Miller, S., Sasaki, Y., Matsumoto, T., Shimonishi, M., Komatsu, T., Terai, T., et al. (2015). Gliotoxin suppresses NF-kappaB activation by selectively inhibiting linear ubiquitin chain assembly complex (LUBAC). ACS Chem. Biol. *10*, 675–681.

Shangary, S., and Wang, S. (2009). Small-molecule inhibitors of the MDM2-p53 protein-protein interaction to reactivate p53 function: a novel approach for cancer therapy. Annu. Rev. Pharmacol. Toxicol. *49*, 223–241.

Sheng, Y., Hong, J.H., Doherty, R., Srikumar, T., Shloush, J., Avvakumov, G.V., Walker, J.R., Xue, S., Neculai, D., Wan, J.W., et al. (2012). A human ubiquitin conjugating enzyme (E2)-HECT E3 ligase structure-function screen. Mol. Cell Proteomics *11*, 329–341.

Shimura, H., Hattori, N., Kubo, S., Mizuno, Y., Asakawa, S., Minoshima, S., Shimizu, N., Iwai, K., Chiba, T., Tanaka, K., et al. (2000). Familial Parkinson disease gene product, parkin, is a ubiquitin-protein ligase. Nat. Genet. *25*, 302–305.

Soucy, T.A., Smith, P.G., Milhollen, M.A., Berger, A.J., Gavin, J.M., Adhikari, S., Brownell, J.E., Burke, K.E., Cardin, D.P., Critchley, S., et al. (2009). An inhibitor of NEDD8-activating enzyme as a new approach to treat cancer. Nature *458*, 732–736.

Stewart, M.D., Ritterhoff, T., Klevit, R.E., and Brzovic, P.S. (2016). E2 enzymes: more than just middle men. Cell Res. *26*, 423–440.

Stieglitz, B., Rana, R.R., Koliopoulos, M.G., Morris-Davies, A.C., Schaeffer, V., Christodoulou, E., Howell, S., Brown, N.R., Dikic, I., and Rittinger, K. (2013). Structural basis for ligase-specific conjugation of linear ubiquitin chains by HOIP. Nature *503*, 422–426.

Strickson, S., Campbell, D.G., Emmerich, C.H., Knebel, A., Plater, L., Ritorto, M.S., Shpiro, N., and Cohen, P. (2013). The anti-inflammatory drug BAY 11-7082 suppresses the MyD88-dependent signalling network by targeting the ubiquitin system. Biochem. J. *451*, 427–437.

Tokunaga, F., Sakata, S., Saeki, Y., Satomi, Y., Kirisako, T., Kamei, K., Nakagawa, T., Kato, M., Murata, S., Yamaoka, S., et al. (2009). Involvement of linear polyubiquitylation of NEMO in NF-kappaB activation. Nat. Cell Biol. *11*, 123–132.

Vassilev, L.T., Vu, B.T., Graves, B., Carvajal, D., Podlaski, F., Filipovic, Z., Kong, N., Kammlott, U., Lukacs, C., Klein, C., et al. (2004). In vivo activation of the p53 pathway by small-molecule antagonists of MDM2. Science *303*, 844–848.

Vogelstein, B., Lane, D., and Levine, A.J. (2000). Surfing the p53 network. Nature *408*, 307–310.

Weisberg, E.L., Schauer, N.J., Yang, J., Lamberto, I., Doherty, L., Bhatt, S., Nonami, A., Meng, C., Letai, A., Wright, R., et al. (2017). Inhibition of USP10 induces degradation of oncogenic FLT3. Nat. Chem. Biol. *13*, 1207–1215.

Welcsh, P.L., and King, M.C. (2001). BRCA1 and BRCA2 and the genetics of breast and ovarian cancer. Hum. Mol. Genet. *10*, 705–713.

Winter, M., Bretschneider, T., Kleiner, C., Ries, R., Hehn, J.P., Redemann, N., Luippold, A.H., Bischoff, D., and Buttner, F.H. (2018). Establishing MALDI-TOF as versatile drug discovery readout to dissect the PTP1B enzymatic reaction. SLAS Discov. https://doi.org/10.1177/2472555218759267.

Yang, Y., Kitagaki, J., Dai, R.M., Tsai, Y.C., Lorick, K.L., Ludwig, R.L., Pierre, S.A., Jensen, J.P., Davydov, I.V., Oberoi, P., et al. (2007). Inhibitors of ubiquitin-activating enzyme (E1), a new class of potential cancer therapeutics. Cancer Res. *67*, 9472–9481.

Zhang, R., Mayhood, T., Lipari, P., Wang, Y., Durkin, J., Syto, R., Gesell, J., McNemar, C., and Windsor, W. (2004). Fluorescence polarization assay and inhibitor design for MDM2/p53 interaction. Anal Biochem. *331*, 138–146.

Zhao, Q., Liu, L., and Xie, Q. (2012). In vitro protein ubiquitination assay. Methods Mol. Biol. *876*, 163–172.

STAR★METHODS

KEY RESOURCES TABLE

REAGENT or RESOURCE	SOURCE	IDENTIFIER
Recombinant Proteins		
Ubiquitin Monomer	Sigma Aldrich	U6253
HOIP	MRC PPU Reagents	Q00987
ITCH	MRC PPU Reagents	Q96J02
MDM2	MRC PPU Reagents	Q96EP0
UBE1	MRC PPU Reagents	P22314
UBA6	MRC PPU Reagents	A0AVT1
UBE2A	MRC PPU Reagents	P49459
UBE2B	MRC PPU Reagents	P63146
UBE2C	MRC PPU Reagents	O00762
UBE2D1	MRC PPU Reagents	P51668
UBE2D2	MRC PPU Reagents	P62837
UBE2D3	MRC PPU Reagents	P61077
UBE2D4	MRC PPU Reagents	Q9Y2X8
UBE2E1	MRC PPU Reagents	P51965
UBE2E2	MRC PPU Reagents	Q96LR5
UBE2E3	MRC PPU Reagents	Q969T4
UBE2G1	MRC PPU Reagents	P62253
UBE2G2	MRC PPU Reagents	P60604
UBE2H	MRC PPU Reagents	P62256
UBE2J1	MRC PPU Reagents	Q9Y385
UBE2J2	MRC PPU Reagents	Q8N2K1
UBE2K	MRC PPU Reagents	P61086
UBE2L3	MRC PPU Reagents	P68036
UBE2N	MRC PPU Reagents	P61088
UBE2O	MRC PPU Reagents	Q9C0C9
UBE2Q1	MRC PPU Reagents	Q7Z7E8
UBE2Q2	MRC PPU Reagents	Q8WVN8
UBE2R1	MRC PPU Reagents	P49427
UBE2R2	MRC PPU Reagents	Q712K3
UBE2S	MRC PPU Reagents	Q16763
UBE2T	MRC PPU Reagents	Q9NPD8
UBE2V1	MRC PPU Reagents	Q13404
UBE2W	MRC PPU Reagents	Q96B02
UBE2Z	MRC PPU Reagents	Q9H832
Chemicals		
2,5-Dihydroxyacetophenone	Bruker Daltonics	8231829
Clomipramine	Sigma Aldrich	C7291
Bendamustine	Sigma Aldrich	B5437
Gliotoxin	Sigma Aldrich	G9893
BAY-11-7082	Sigma Aldrich	B5556
PYR-41	Sigma Aldrich	N2915
PR-619	Millipore	662141
Compound library of 1430 FDA approved drugs	DDU	

CONTACT FOR REAGENT AND RESOURCE SHARING

Further information and requests for resources and reagents should be directed to and will be fulfilled by the corresponding authors Virginia De Cesare (v.decesare@dundee.ac.uk), Matthias Trost matthias.trost@ncl.ac.uk). PYR41 and Nutlin3A were kindly provided by Sara Buhrlage, PhD, Dana Farber Cancer Institute. Compound1 was kindly provided by Satpal Virdee, PhD, MRC-PPU Dundee. Plasmids generated at the University of Dundee for the present study are available to request on our reagents website (https://mrcppureagents.dundee.ac.uk/).

EXPERIMENTAL MODEL AND SUBJECT DETAILS

Ubiquitin monomer, BSA, Tris, DTT and Gliotoxin were purchased from Sigma-Aldrich. MALDI TOF MS materials (targets, matrix and protein calibration mixture) were purchased from Bruker Daltonics (Bremen, Germany). PYR41 and Nutlin3A compounds were kindly provided by Sara Buhrlage (Dana-Farber Cancer Institute) and Compound-1 was kindly provided by Satpal Virdee (MRC PPU, Dundee).

E1, E2, ITCH and HOIP E3 Enzyme Expression and Purification

^{15}N-labelled ubiquitin was produced as described in Ritorto et al (Ritorto et al., 2014). Human recombinant 6His-tagged UBE1 was expressed in and purified from Sf21 cells using standard protocols. Human E2s were all expressed as 6His-tagged fusion proteins in BL21 cells and purified via their tags using standard protocols. Briefly, BL21 DE3 codon plus cells were transformed with the appropriate constructs (see table below), colonies were picked for overnight cultures, which were used to inoculate 6 x 1L LB medium supplemented with antibiotics. The cells were grown in Infors incubators, whirling at 200 rpm until the OD_{600} reached 0.5 – 0.6 and then cooled to 16°C – 20°C. Protein expression was induced with typically 250 μM IPTG and the cells were left over night at the latter temperature. The cells were collected by centrifugation at 4200 rpm for 25min at 4°C in a Beckman J6 centrifuge using a 6 x 1 L bucket rotor (4.2). The cells were resuspended in ice cold lysis buffer (50 mM Tris-HCl pH 7.5, 250 mM NaCl, 25 mM imidazole, 0.1 mM EGTA, 0.1 mM EDTA, 0.2 % Triton X-100, 10 μg/ml Leupeptin, 1 mM PefaBloc (Roche), 1mM DTT) and sonicated. Insoluble material was removed by centrifugation at 18500 xg for 25 min at 4°C. The supernatant was incubated for 1 h with Ni-NTA-agarose (Expedeon), then washed five times with 10 volumes of the lysis buffer and then twice in 50 mM HEPES pH 7.5, 150 mM NaCl, 0.015% Brij35, 1 mM DTT. Elution was achieved by incubation with the latter buffer containing 0.4M imidazole or by incubation with Tobacco Etch Virus (TEV) protease (purified in house). The proteins were buffer exchanged into 50 mM HEPES pH 7.5, 150 mM NaCl, 10% glycerol and 1 mM DTT and stored at -80°C. HOIP (697-1072) DU22629 and Itch (DU11097) ligases were expressed in BL21 cells as GST-tagged fusion proteins, purified via their tag and collected by elution (GST-Itch) or by removal of the GST-tag on the resin (HOIP).

MDM2 E3 Enzyme Expression and Purification

pGex-Mdm2 [DU 43570] was expressed in BL21 (DE3) *E. coli* cells grown in LB media containing 100 μg/ml ampicillin. Cells were induced with 250 μM isopropyl beta-D-1-thiogalactopyranoside (IPTG) at an OD600 of 0.6-0.8 and grown for 16 hours at 15°C. Cells were pelleted and resuspended in 50 mM Tris-HCl pH 7.5, 250 mM NaCl, 1 % Triton, 1 mM EDTA, 1 mM EGTA, 0.1 % 2-mercaptoethanol, 1 mM Pefabloc, 1 mM benzamidine. Cell lysis was carried out by sonication. After being clarified through centrifugation, bacterial lysate was incubated with Glutathione Agarose (Expedeon) for 2 hours at 4°C. The resin bound proteins were washed extensively with Wash buffer (50 mM Tris-HCl pH 7.5, 250 mM NaCl, 0.1 mM EGTA, 0.1 % 2-mercaptoethanol), before being eluted with wash buffer containing 20 mM Glutathione. The purified proteins were dialysed into storage buffer, flash frozen and stored at -80°C.

Plasmids generated at the University of Dundee for the present study are available to request on our reagents website (https://mrcppureagents.dundee.ac.uk/).

METHOD DETAILS

E1/E2/E3 Assay

The E2-E3 reaction consists of recombinant E1 (100 nM), E2 conjugating enzyme (125-250 nM), E3 ligases (250-500 nM) and 0.25 mg/mL BSA in 10 mM HEPES pH 8.5, 10 mM $MgCl_2$ and 1 mM ATP in a total volume of 5 μl. Assays were performed by dispensing 2.5 μL of enzyme solution into round bottom 384-well plates (Greiner, Stonehouse, UK). Plates were centrifuged at 200 xg and the reactions were incubated at 37°C for 30 min. Reactions were initiated by the addition of 2.5 μL substrate solution containing 10 μM ubiquitin in 5 mM HEPES pH 8.5. For enzyme titration and time course experiments E2/E3 ligases concentrations ranged from 125 nM to 1000 nM with a maximum reaction time of 120 min. Plates were incubated at 37°C for typically 20-60 min (depending on the activity of E3 ligase used) before being quenched by the addition of 2.5 μL of a 10 % (v/v) trifluoroacetic acid (TFA) solution. Controls – with only DMSO - where placed on column 23. For the enzyme inactivated controls in columns 24, 2.5 μL of 10 % TFA was manually dispensed prior to addition of the enzyme solution by XRD-384 Automated Reagent Dispenser (FluidX). 1.05 μl of each reaction were spiked with 150 nl (4 μM) of ^{15}N-labelled ubiquitin (average mass 8,659.3 Da) and 1.2 μl of 7.6 mg/ml 2,5-dihydroxyacetophenone (DHAP) matrix (prepared in 375 ml ethanol and 125 ml of an aqueous 25 mg/ml diammonium hydrogen citrate).

For E2 activity assays, we pre-incubated the Ube1 activating enzyme (100 nM) with 27 E2 conjugating enzymes at 1000 nM, 500 nM and 250 nM and stopped the reaction with 2% final TFA at different time points.

Compound Library Spotting and Inhibitor Screening

We used a library of 1430 FDA approved compounds from various commercial suppliers with validated biological and pharmacological activities. For single concentration screening 5 nL of 10 mM compound solution in DMSO was transferred into HiBase Low Binding 384-well flat bottom plates (Greiner bio-one) to give a final screening concentration of 10 μM. Columns 23 and 24 were reserved for uninhibited and inhibited controls respectively. The uninhibited control contained 5 nL DMSO but no compound, whereas the inhibited control contained 5 nL PR-619 but the enzyme was inactivated by pre-treatment with 1.0 % TFA. All compounds and DMSO were dispensed using an Echo acoustic dispenser (Labcyte, Sunnyvale, USA). For all HTS assays the final DMSO concentration was 0.1 %. For concentration response curves of known HOIP, MDM2 and ITCH inhibitors, a threefold serial dilution was prepared from 10 mM compound solutions in DMSO in 384-well base plates V-Bottom (Labtech). 100 nL of compound was transferred into 384-well round bottom low binding plates using a Mosquito Nanoliter pipetter (TTP Labtech, Melbourn, UK), giving a final concentration range between 100 μM and 100 nM.

Target Spotting and MALDI Mass Spectrometry Analysis

1,536-well AnchorChip MALDI targets (Bruker, Bremen Germany) were cleaned using 30% acetonitrile and 0.1% TFA and dried under a gentle flow of pure nitrogen. 200 nL matrix/assay mixture was spotted onto the AnchorChip Plates using a Mosquito nanoliter dispenser (TTP Labtech, Hertfordshire, UK). Spotted targets were air dried prior to MALDI TOF MS analysis. 0.25 μl of the resultant mixture was then spotted onto a 1,536 microtiter plate MALDI anchor target (Bruker, Bremen, Germany) using a Mosquito liquid handling robot (TTP Labtech, Melbourn, UK).

All samples were acquired on a Rapiflex MALDI TOF mass spectrometer (Bruker Daltonics, Bremen, Germany) high resolution MALDI TOF MS instrument with Compass for flexSeries 2.0. Reflector mode was used with optimized voltages for reflector 1 (20.82 kV), reflector 2 (1.085 kV) and reflector 3 (8.8 kV), ion sources (Ion Source 1: 20.0 kV, PIE 2.35 kV) and Pulsed Ion Extraction (500 ns). Matrix suppression has been set in Deflection mode to suppress matrix components up to 6560 *m/z.* Samples were run in automatic mode (AutoXecute, Bruker Daltonics) using the 1,536 spots AnchorChip. Ionization was achieved by a 10-kHz smartbeam-II solid state laser (run at 5 kHz) with Laser Fuzzy Control switched off, initial Laser Power set on “from Laser Attenuator” and accumulation parameters set to 4000 satisfactory shots in 500 shot steps. Movement parameters have been set on “Walk on Spot”. Spectra were accumulated by FlexControl software (version 4 Build 9), processed using FlexAnalysis software (version 4 Build 9) and the sophisticated numerical annotation procedure (‘SNAP’) peak detection algorithm, setting the signal-to-noise threshold at 5. Internal calibration was performed using the ubiquitin peak ($[M+H]^+$ average = 8,659.3).

Data Analysis

A modified method for data acquisition was developed for FlexAnalysis Software version 4, using the SNAP algorithm. For area calculation, the complete isotopic distribution was taken into account. Data output was exported as a.csv file using FlexAnalysis series 4.0 (Build 24) Batch Process (Compass for flexseries 2.0). An in-house script has been used to extract - from the original csv output file - the data of interest. The script selects the area values of the light and the heavy ubiquitin and reports them in a grid with the same MALDI target geometry and sample positions within a txt file. Data are further analysed in Microsoft Excel, where plotting of graphs and IC50 calculation have been performed on Prism 7 software for windows, version 7.02.

For HTS data analysis, data was expressed as Activity % value for each test compound as follows:

$$Activity\ \% = 100 - \left[\frac{\left(\frac{light}{heavy}test\right) - \left(\mu\ \frac{light}{heavy}n\right)}{\left(\mu\ \frac{light}{heavy}p\right)}\right]$$

where $\frac{light}{heavy}$ *test* is the light ubiquitin signal normalized to the heavy ubiquitin signal associated with the test compound, $\mu\frac{light}{heavy}n$ is the average of the light ubiquitin signal normalized to the heavy ubiquitin in the no inhibition signal positive controls (reaction in presence of DMSO only) and $\mu\frac{light}{heavy}p$ is the is the average of the light ubiquitin signal normalized to the heavy ubiquitin of maximum effect negative control wells (reaction where 2% TFA final has been added before the addition of substrate)

The performance of the assay on each screening plate was evaluated using internal controls to determine robust Z’ values, which were calculated as follows:

$$Z' = 1 - \left(\frac{3(\sigma_p + \sigma_n)}{|\mu_{p-}\ \mu_n|}\right)$$

Where the means (μ) and standard deviations (σ) of both the positive (p) and negative (n) controls are reported.

QUANTIFICATION AND STATISTICAL ANALYSIS

MALDI-TOF data output was exported and analysed by an in-house script as reported in the Data Analysis Paragraph. Data were further analysed in Microsoft Excel, where plotting of graphs and IC50 calculation have been performed on Prism 7 software for windows.

For linearity curves and HTS data analysis, data was expressed as Activity % value for each test compound following the formula reported in Data Analysis Paragraph. For IC50s calculation, duplicate experiments were averaged and the standard deviation of the mean were calculated using the GraphPad Prism software with a built-in analysis, nonlinear regression (curve-fit), variable slope (four parameters) curve to determine IC50 values.

Cell Chemical Biology, Volume *25*

Supplemental Information

The MALDI-TOF E2/E3 Ligase Assay as Universal Tool for Drug Discovery in the Ubiquitin Pathway

Virginia De Cesare, Clare Johnson, Victoria Barlow, James Hastie, Axel Knebel, and Matthias Trost

Supplementary Data

Table S1: Initial reaction/Instantaneous rate at 5 min of MDM2, ITCH and HOIP (*related to Figure 1*)

Instantaneous Rate at *t* = 5 min

**µM Ub**	**12.5**	**6.25**	**3.125**	
MDM2	0.61	0.24	0.30	µmol/min
ITCH	0.41	0.34	0.30	µmol/min
HOIP	1.85	1.28	0.94	µmol/min

Table S2: List of E1 Activating enzymes, E2 conjugating enzymes and E3 ligases used in this study (*related to Figure 2 and STAR methods*)

		Name	Uniprot Accession Number	Alter-native Name	Tag	Domain	Host	E2/E3 active pair	Activity
E1	1	UBE1	P22314	-	His	full length	*S. frugiperda*	-	-
	2	UBA6	A0AVT1	-	His	full length	*S. frugiperda*	-	-
E2 Conjugating Enzyme	3	UBE2A	P49459	-	His	2-152	*E. coli*	-	-
	4	UBE2B	P63146	-	His	full length	*E. coli*	-	-
	5	UBE2C	O00762	UbcH10	-	full length	*E. coli*	ITCH	Low
	6	UBE2D1	P51668	UbcH5a	-	full length	*E. coli*	MDM2-ITCH-HOIP	High
	7	UBE2D2	P62837	UbcH5b	His	2-147	*E. coli*	MDM2-ITCH-HOIP	High
	8	UBE2D3	P61077	UbcH5c	His	2-147	*E. coli*	MDM2-ITCH-HOIP	Moderate-High
	9	UBE2D4	Q9Y2X8	UbcH5D	-	full length	*E. coli*	MDM2-ITCH-HOIP	Moderate-High
	10	UBE2E1	P51965	UbcH6	His	full length	*E. coli*	MDM2-ITCH-HOIP	Low-Moderate
	11	UBE2E2	Q96LR5	UbcH8	His	full length	*E. coli*	MDM2-HOIP	Moderate
	12	UBE2E3	Q969T4	UbcH9	His	full length	*E. coli*	MDM2-HOIP	Moderate
	13	UBE2G1	P62253	Ubc7	His	full length	*E. coli*	-	-
	14	UBE2G2	P60604	-	His	full length	*E. coli*	-	-
	15	UBE2H	P62256	Ubc8	His	full length	*E. coli*	-	-
	16	UBE2J1	Q9Y385	Ubc6e	His	full length	*E. coli*	-	-
	17	UBE2J2	Q8N2K1	-	-	full length	*E. coli*	-	-
	18	UBE2K	P61086	Ubc1	His	full length	*E. coli*	MDM2-ITCH-HOIP	Low
	19	UBE2L3	P68036	UbcH7	-	full length	*E. coli*	ITCH-HOIP	Moderate-High
	20	UBE2N	P61088	Ubc13	His	full length	*E. coli*	-	-
	21	UBE2O	Q9C0C9	E2-230K	His	552-1292	*E. coli*	-	Basal Activity
	22	UBE2Q1	Q7Z7E8	UBE2Q	His	full length	*E. coli*	-	Basal Activity
	23	UBE2Q2	Q8WVN8	-	His	full length	*E. coli*	-	Basal Activity
	24	UBE2R1	P49427	-	His	2 – 236	*E. coli*	-	-
	25	UBE2R2	Q712K3	Ubc3B	-	full length	*E. coli*	-	-
	26	UBE2S	Q16763	E2-EPF	His	full length	*E. coli*	-	-
	27	UBE2T	Q9NPD8	HSPC150	His	full length	*E. coli*	-	-
	28	UBE2V1	Q13404	Uev1A	His	full length	*E. coli*	-	-
	29	UBE2W	Q96B02	-	His	full length	*E. coli*	-	-
	30	UBE2Z	Q9H832	USE1	His	full length	*E. coli*	-	-
E3 Ligase	31	MDM2	Q00987	-	GST	7 - 497	*E. coli*	-	-
	32	ITCH	Q96J02	-	GST	full length	*E. coli*	-	-
	33	HOIP	Q96EP0	-	-	697-1072	*E. coli*	-	-

E. coli, *Escherichia coli*; *S. frugiperda*, *Spodoptera frugiperda*; *Low (≤50% Activity after 2h incubation time); Moderate (≥50% and ≤80% Activity after 2h incubation time); High 100% Activity after 2h Incubation time

Table S3| IC50 calculation of six E1, E2 or E3 inhibitors (*related to Figure 4*)

IC50 (µM)

	PR619	Nutlin3A	Gliotoxin	BAY117082	PYR41	Compound1
MDM2	0.6	Ambiguous	0.5	2.4	3.1	1.2
ITCH	0.4	Ambiguous	30.6	25.9	11.3	2.2
HOIP	0.2	Ambiguous	2.8	2.9	5.7	2.3

Table S4| Hill Slopes values for calculated IC50s (*related to Figure 4*)

Hill Slope Values

	PR619	Nutlin3A	Gliotoxin	BAY117082	PYR41	Compound1
MDM2	1.89	-	2.18	3.5	2.23	0.67
ITCH	3.14	-	5.12	1.23	1.22	0.78
HOIP	7.8	-	2.01	2.48	3.73	1.14

Table S5| Z-Prime scores (*related to Figure 5*)

	Plate				
	A	B	C	D	E
MDM2	0.64	0.57	0.63	0.62	0.55
ITCH	0.67	0.64	0.73	0.83	0.79
HOIP	0.60	0.56	0.59	0.67	0.62

Figure S1

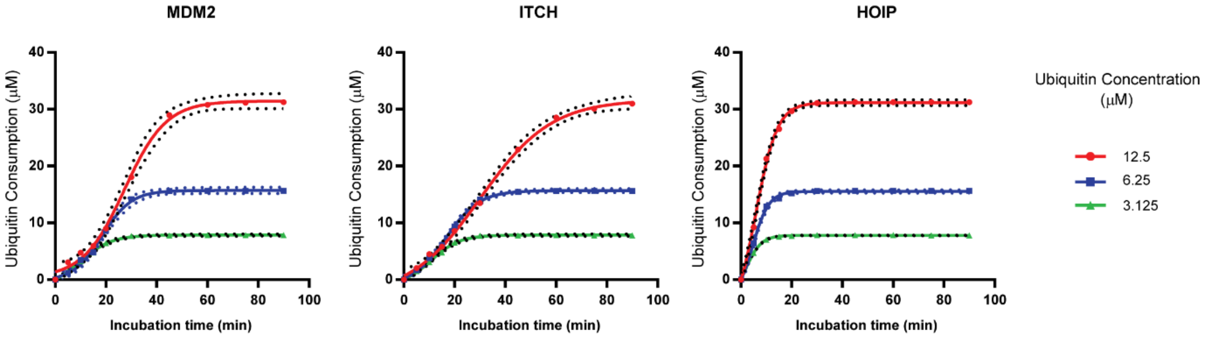

Figure S1: Substrate consumption over time of MDM2, ITCH and HOIP. ***(**related to Figure 1**)*** Rate of disappearance of ubiquitin at different starting concentrations were measured for MDM2, ITCH and HOIP. Ubiquitin consumption (Ubiquitin μM t2 - Ubiquitin μM t1)) was plotted over time. Data are represented as mean +/- standard deviation.

Figure S2

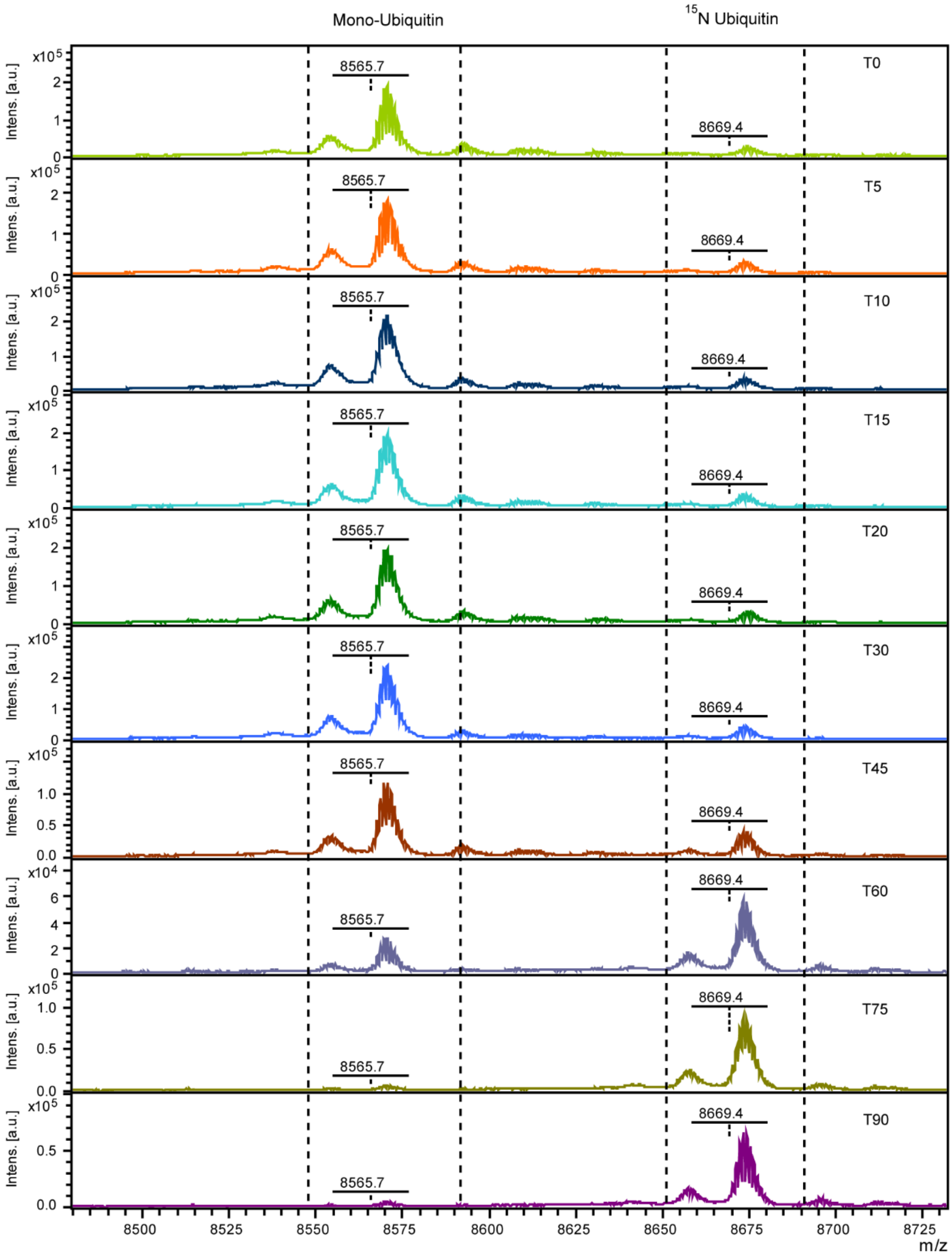

Figure S2: E2-E3 MALDI-TOF assay representative spectra (*related to Figure 1*). MALDI-TOF spectra of MDM2 activity at different time points (indicated as T0, T5 etc.) are reported. MDM2 was incubated with 50 nM E1, 250 nM E2D1, 1 mM ATP, 12.5 µM ubiquitin at 37°C and terminated at the indicated time points by addition of 2.5 µl of 10% (v/v) trifluoroacetic acid (TFA). The samples were spotted and analysed by high mass accuracy MALDI TOF MS as indicated in Material and Methods.

Figure S3

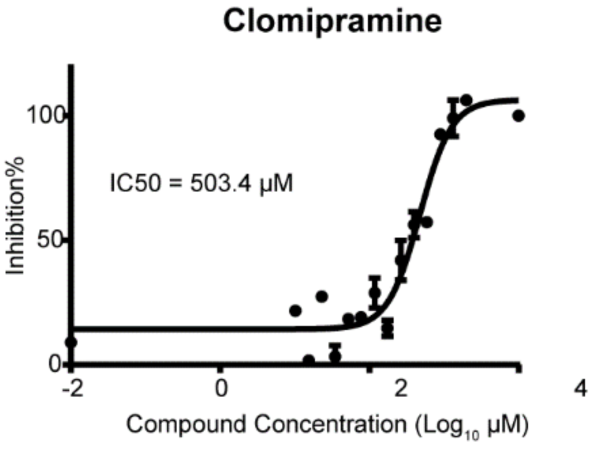

Figure S3: Clomipramine inhibits ITCH at high concentration *(related to Figure 4).* Clomipramine was tested against ITCH in a dose-response curve with final concentration values between 2mM and 7 µM. The results indicated an IC50 of 503.4 µM. Data are represented as mean +/- standard deviation.

Figure S4

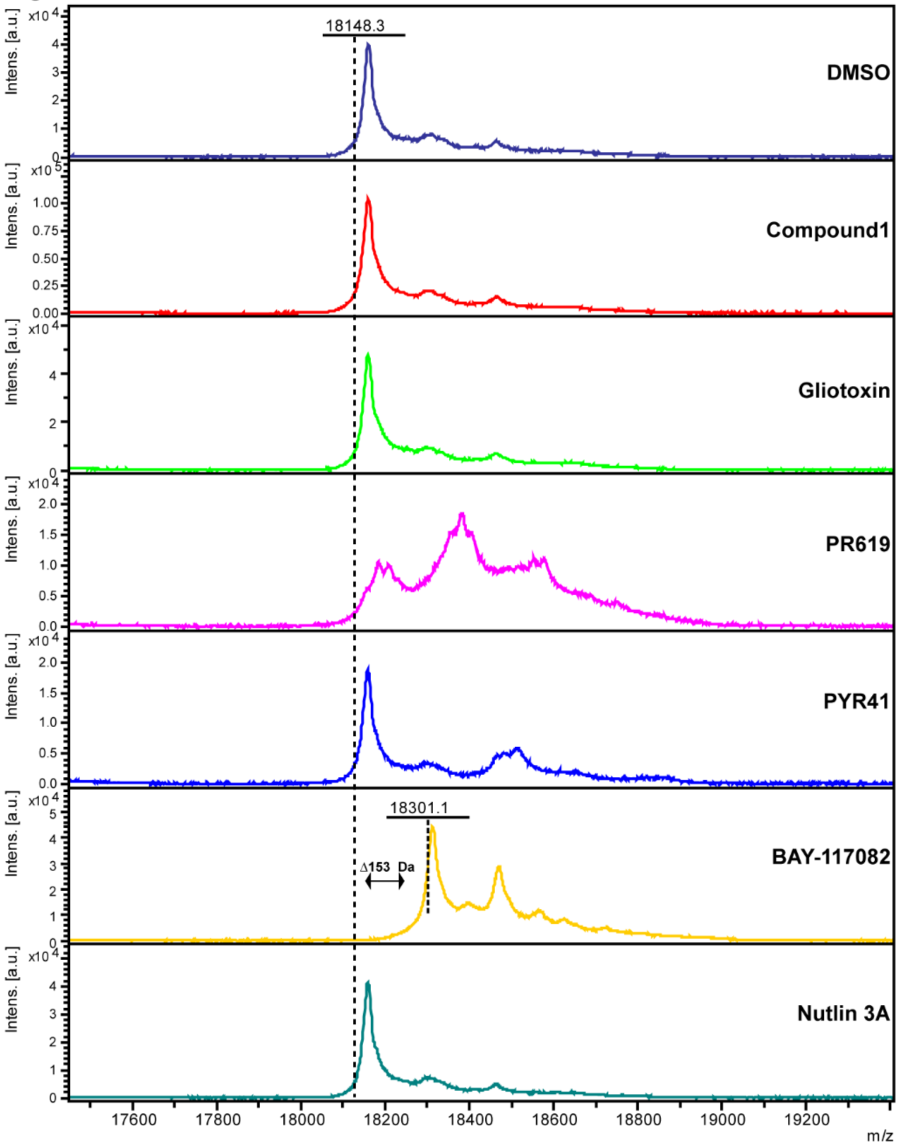

Figure S4:Covalent modifications of E2 enzymes by inhibitors *(related to Figure 4)*.
UBE2L3 (3 µM) was incubated for 30 min at room temperature with or without the indicated inhibitors (100 µM) and subjected to MALDI–TOF-MS. Incubation with BAY 11-7082 increased the molecular mass of UBE2L3 by 153 Da. PR619 extensively modified UBE2L3 indicated several PR619 adducts.

Figure S5

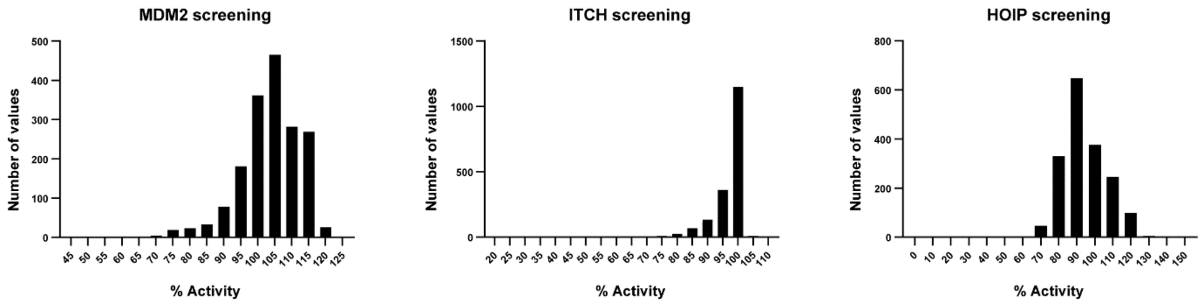

Figure S5: Frequency distribution of HTS data of MDM2, ITCH and HOIP *(related to Figure 5)*.
Bars report number of values falling within the reported % Activity.

Figure S6

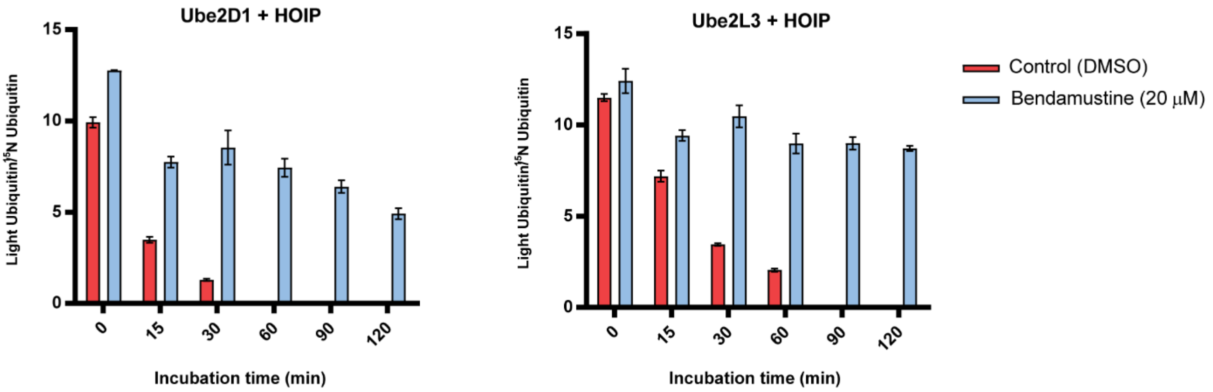

Figure S6: Bendamustine inhibits HOIP independently from the E2 conjugating enzyme *(related to Figure 6).*
Bendamustine (20 µM) was tested against HOIP coupled with Ube2D1 and Ube2L3 over time. Bendamustine inhibits HOIP independently from the E2 in use. Data are represented as mean +/- standard deviation.
